# Linker-Assisted CdS-TiO_2_ Nanohybrids as Reusable Visible Light Photocatalysts for the Oxidative Hydroxylation of Arylboronic Acids

**DOI:** 10.1021/acs.joc.2c02964

**Published:** 2023-03-17

**Authors:** Willber D. Castro-Godoy, Luciana C. Schmidt, Diego Flores-Oña, Julia Pérez-Prieto, Raquel E. Galian, Juan E. Argüello

**Affiliations:** † INFIQC-CONICET-UNC, Departamento de Química Orgánica, Facultad de Ciencias Químicas, Universidad Nacional de Córdoba, Ciudad Universitaria, X5000HUA Córdoba, Argentina; ‡ CENSALUD-UES, Departamento de Química, Física y Matemática, Facultad de Química y Farmacia, Universidad de El Salvador, Final Av. Mártires y Héroes del 30 de Julio, San Salvador 1101, El Salvador; § Instituto de Tecnología Química, Universitat Politècnica de València-Consejo Superior de Investigaciones Científicas, Avenida de los Naranjos s/n, 46022 Valencia, Spain; ∥ Institute of Molecular Science (ICMol), University of Valencia, Catedrático José Beltrán 2, Paterna 46980, Valencia, Spain; ⊥ Facultad de Ingeniería Química Universidad Central de Ecuador, Rither y Bolivia, Ciudadela Universitaria, 170521 Quito, Ecuador

## Abstract

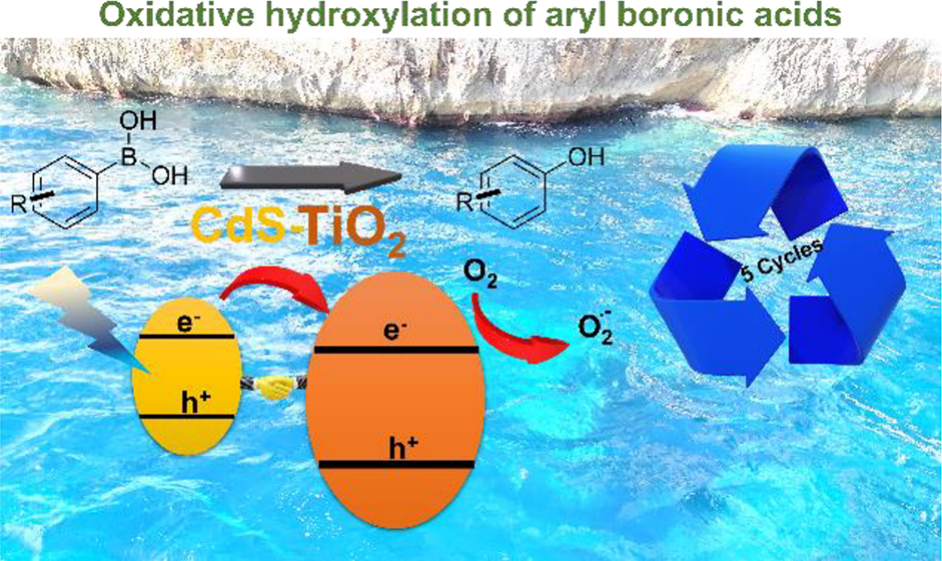

A variety of phenols have been obtained in aqueous media with moderate to excellent chemical yields (≤100%) by using arylboronic acids and esters as substrates, a robust CdS-TiO_2_ nanohybrid as a heterogeneous photocatalyst, visible light irradiation (467 nm), and an O_2_-saturated atmosphere. The nanohybrid was prepared through a linker-assisted methodology that uses mercapto alkanoic acids as the organic linkers. The nanohybrid showed improved photocatalytic activity in the hydroxylation of substituted arylboronic acids and phenyl boronic esters compared with that of pristine CdS quantum dots. The nanohybrid can be reused in up to five photocatalytic cycles with ∼90% of its outstanding activity preserved.

## Introduction

Visible light-promoted photoredox catalysis is considered a powerful tool in organic synthesis.^1−5^ Despite the significant advances in the field of photocatalysis in recent years, new heterogeneous photocatalysts are still lacking, especially those dispersible and stable in aqueous media. Moreover, visible light is an innocuous reagent that can generate reactive species, prevent waste formation, and be obtained from renewable sources.^6−9^

Zero-dimensional semiconductor materials, usually known as quantum dots (QDs), have exceptional photophysical properties, such as broad absorption spectra, narrow emission spectra, and high photostability compared to those of conventional organic fluorophores.^10,11^ Their optical and electronic properties are size-dependent and can be tuned as required, making these materials relevant for their application in medicine, biology, technology, and, more recently, photocatalysis.^12−15^ Although TiO_2_ semiconductor nanoparticles have been extensively used in photocatalysis for dye degradation and organic synthesis due to their high catalytic activity, chemical stability, and low cost, their use is restricted due to their narrow spectral response (band gap of ∼3.2 eV).^16^ The coupling of chalcogenide QDs, such as CdSe or CdS, to the TiO_2_ surface gives rise to nanohybrids,^17−19^ which improve the photocatalytic properties of TiO_2_ due to the extension of light absorption toward the visible region and increase the charge separation while favoring the reusability of the QDs in aqueous media. Several strategies have been proposed to bind chalcogenide QDs to TiO_2_ nanoparticles;^20−22^ however, it is still necessary to design a strategy to link both types of nanoparticles properly and to bring about a robust nanohybrid for organic synthesis in aqueous media. Bifunctional ligands, such as mercapto alkanoic acids (HS-R-COOH), are good candidates for binding chalcogenide QDs to the TiO_2_ surface and have previously been used to tether QDs to other nanostructures.^23,24^

Phenolic compounds are known to act as natural antioxidants and have extensive industrial applications.^25−28^ Today, the oxidative hydroxylation of arylboronic acids represents a convenient alternative for preparing them compared to conventional phenol synthesis methods, such as the nucleophilic substitution of aryl halides for a hydroxyl group and diazotization of aromatic amines followed by aqueous hydrolysis and C–H aryl ring oxidation. These methods are not usually very compatible with easily oxidizable functional groups, and the starting materials are highly toxic, require special storage, or are complex to handle. Although phenols can be obtained by using strong oxidizing^29−32^ and reducing^33^ agents, these approaches usually use stoichiometric amounts of reagents or require high temperatures. In the past decade, catalytic oxidative hydroxylation of boronic acids has been exhaustively studied and some examples include the use of quinones,^34,35^ flavin derivatives,^36^ magnetic CuFe_2_O_4_, Fe_2_O_3_@SiO_2_, Cu_2_O, and Cu nanoparticles,^37−39^ and Pd or Cu complexes.^40,41^ More recently, visible light photoredox catalysis with organometallic complexes,^42^ organic molecules,^43−45^ and semiconductor nanoparticles^46,47^ has been developed. Thus, CdSe QDs have been used as photoredox catalysts (0.08 mol %) for the aerobic oxidation of boronic acids under visible light in anhydrous acetone by using oxygen as the oxidant, thereby providing good to high product yields and turnover numbers (TONs) of >6200. Although the reaction was efficient for aromatic and aliphatic boronic acids, the yield drastically decreased for boronic esters (16%). In addition, the phenol yield was high (≤88%) for 4-cyano-phenylboronic acid, and although the reusability of the photocatalyst was limited to three cycles, the yield was still good (62%) after irradiation for 96 h. The authors proposed that the reaction occurred via an initial fast electron transfer (350 ps) from the CdSe QD to the Lewis acid–base adduct between boronic acid and a tertiary amine, followed by a hole transfer from the QD to the amine; nevertheless, the mechanism is still being studied.^48^

Supported semiconductor nanoparticles on the TiO_2_ surface could be excellent candidates for overcoming the limitations mentioned above in the oxidative hydroxylation of aromatic boronic acids in aqueous media. It is desirable to improve not only the phenol yield but also the reusability of the nanocatalyst to broaden the scope of the reaction.

The photocatalytic quantum efficiency in a semiconductor system relies on the improvement of charge separation to delay the hole–electron recombination process, by coupling semiconductors with appropriate valence and conduction bands (VB and CB, respectively). As demonstrated in previous reports, the charge separation in CdS QDs can greatly improve upon coupling with TiO_2_ nanoparticles.^49,50^ Most of these assemblies have been used for the photocatalytic degradation of organic dyes.^24,51,52^

**Scheme 1:**
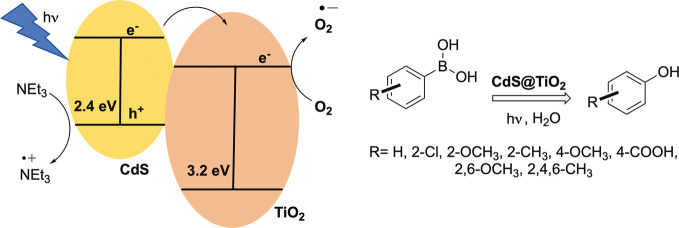
General Procedure for the Oxidative Hydroxylation of Aryl Boronic Acids and Esters Using CdS-TiO_2_ Nanohybrids

We illustrate here the exceptional photocatalytic activity of the linker-assisted assembly of CdS/TiO_2_ nanoparticles in the oxidative hydroxylation of a battery of aryl boronic acids and esters (Scheme 1). CdS QDs were chosen as light-absorber semiconductors in the assembly because they present an appropriate band gap (∼2.4 V) to allow visible light excitation^53^ and suitable band edge positions for the photocatalytic redox reaction (VB of −6.1 eV and CB of −3.7 eV)^54^ that match the energy of the VB and CB of TiO_2_ reported as −7.1 and −3.95 eV, respectively (band gap of 3.2 eV).^55^ Moreover, the appropriate binding of CdS QDs to the TiO_2_ surface by mercapto alkanoic acids, such as mercaptopropionic (MPA) and mercaptosuccinic (MSA) acids, was successfully implemented. This strategy ensures the charge transport between both semiconductors and the photocatalyst can be reused up to five cycles with a high chemical yield.

## Results and Discussion

### Synthesis and Characterization of the CdS QDs

The synthesis of water-dispersible CdS QDs was carried out by following a previously described procedure with some modifications.^56^ Briefly, cadmium chloride was used as the Cd precursor, and MPA or MSA acids were used as sources of sulfur and organic ligands. The reaction was carried out in aqueous basic media, in the presence of hydrogen peroxide, under reflux and a nitrogen atmosphere. Different aliquots were pipetted out at 1, 2, and 24 h (see the Supporting Information for more details); the CdS@MSA and CdS@MPA QDs obtained were purified by centrifugation, and the pellet was dried under vacuum for further optical characterization.

The ultraviolet–visible (UV–vis) absorption spectra of the different aliquots of CdS@MSA and CdS@MPA QDs (1.2 mg in 5 mL of an aqueous solution) were recorded. The excitonic peaks shifted to longer wavelengths as the reaction time increased: from 415 to 440 nm (Figure S1a) and from 365 to 460 nm (Figure S1b) for CdS@MSA and CdS@MPA QDs, respectively. These QDs exhibited low photoluminescence, with a Stokes shift that depended on the QD size (Figure S1c,d). The QDs obtained after 24 h showed the highest colloidal stability, and they were chosen for the microscopic characterization and for the anchoring to the TiO_2_ nanoparticle surface.

The morphology of pristine CdS QDs was evaluated with high-resolution transmission electron microscopy (HRTEM). Both QDs presented a quasi-spherical shape that was ∼6–7 nm in diameter (Figure S2). Some nanoparticle aggregation was observed in the TEM images for the thiol-coated QDs, as previously reported for hydrophilic thiol-coated CdSe QDs and *N*-acetyl-l-cysteine-capped CdTe QDs.^57,58^ A close inspection of the lattice fringes (Figure S2b,d) revealed the presence of various crystalline planes. In the case of CdS@MSA, lattice distances (*d*) of 0.296 and 0.210 nm were observed, which match the interplanar spacing of the (200) and (220) crystal planes of cubic CdS QDs, respectively. However, for CdS@MPA, lattice distances of 0.335 and 0.209 nm indicated a preferential growth of (111) and (220) crystal planes of cubic CdS QDs, respectively.^59−61^ These results agreed with the X-ray diffraction analysis for both QDs (Figure S3); the XRD spectra exhibited three main peaks at 26.6°, 44.0°, and 51.9°, which corresponded to the (111), (220), and (311) crystalline planes of the CdS cubic phase, respectively. A small contribution of the hexagonal phase of CdS was observed at 24.9° and 28.2°, in the case of CdS@MSA (Figure S3a), which evidenced that the nature of the organic capping can regulate the specific adsorption of the ligand on preferential crystalline faces.^62^

### Synthesis and Characterization of the CdS-TiO_2_ Nanohybrid

**Figure 1:**
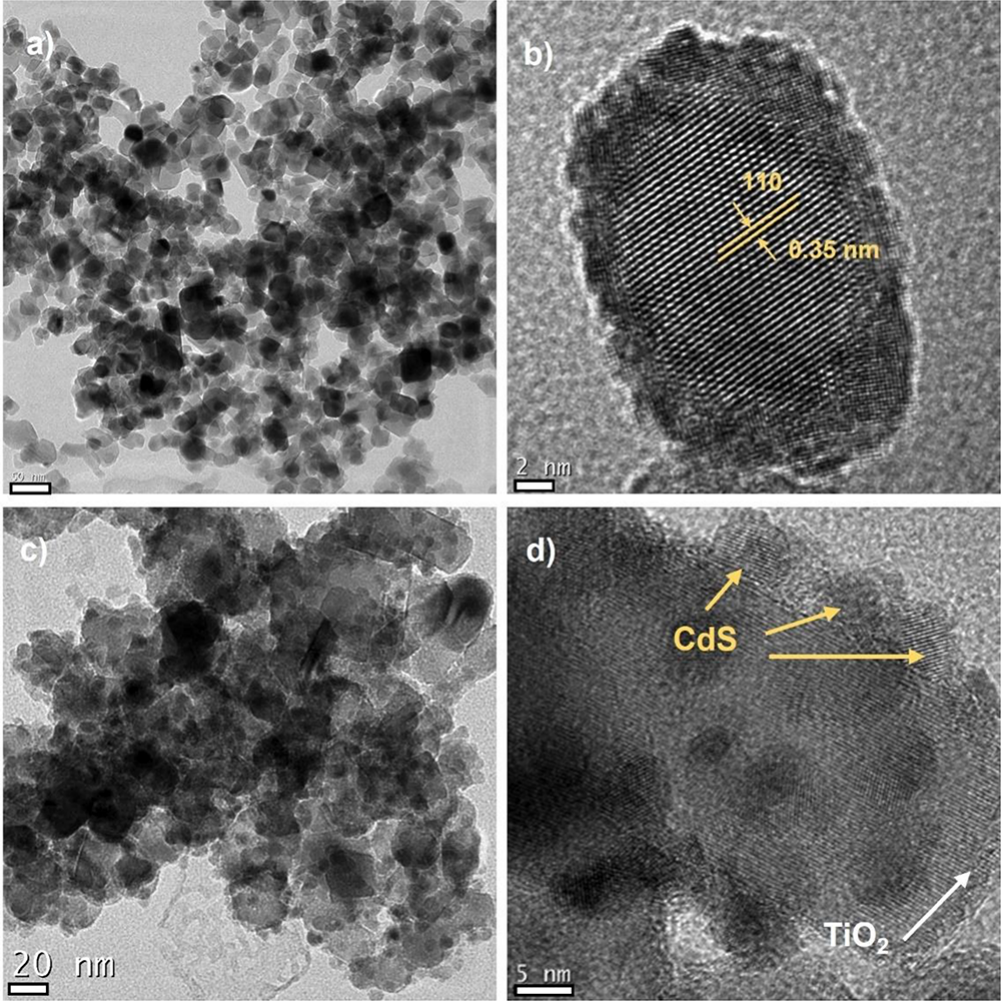
HRTEM images of (a and b) Degussa TiO_2_ and (c and d) the CdS@MPA-TiO_2_ hybrid. Scale bars of (a) 50, (b) 2, (c) 20, and (d) 5 nm.

Commercially available Degussa TiO_2_ Aeroxide P25 was used for the preparation of the nanohybrid, and its morphology was characterized by HRTEM. Irregular cuboid-shaped nanoparticles with a length of ∼25 nm and a width of ∼17 nm were detected (Figure 1a). The lattice fringes of a Degussa TiO_2_ nanoparticle were clearly observed in Figure 1b with a lattice spacing of 0.350 nm ascribed to the (101) crystal plane of the anatase phase.^63−65^

It has previously been reported that bifunctional organic ligands on the surface of chalcogenide QDs can anchor to the TiO_2_ nanoparticle surface, thus resulting in linker-assisted assemblies whose efficiency for electron transfer depends on the QD size, interfacial energy, and linker properties.^66^ Several strategies have been developed. Method 1 involves the functionalization of a substrate with a bifunctional linker molecule, followed by the binding of the QDs to the anchored ligands. Method 2 consists of the attachment of the QD capped with a bifunctional ligand to a bare TiO_2_ surface. Method 3 combines the linker-assisted assembly with *in situ* manufacturing.^66^ In this report, a modified linker-assisted assembly via method 2 was used. Several parameters were analyzed, such as pH (7.5, 8.5, 10, and 11.5), temperature for the binding process (25 and 50 °C), QD concentration (5%, 10%, 20%, 30%, 40%, and 50%), and the amount of additional bifunctional ligands (MSA or MPA) used to facilitate the binding of both semiconductors. Briefly, the CdS QDs synthesized with the bifunctional mercapto alkanoic acids (CdS@MSA or CdS@MPA QDs) were attached to the TiO_2_ surface (QD, 25%) by using additional bifunctional ligands and a basic medium (NaOH). The mixture was sonicated in an ultrasound bath at 50 °C for 1 h, and the mixture was centrifuged to provide a precipitate, which was first washed to remove the unreacted materials and then dried under vacuum for further use (Figure S4). Both assemblies were built, but only CdS@MPA-TiO_2_ was isolated as a solid product, which facilitated its characterization and use in the photocatalytic assays. It is noteworthy that the absence of QDs in the supernatant evidenced the proper binding of the CdS QDs to the TiO_2_ surface. The addition of bifunctional organic ligands (modified method 2) was crucial to guarantee the successful attachment of the CdS QDs to the TiO_2_ surface.^66^ HRTEM images of the assembly (see panels c and d of Figure 1 for CdS@MPA-TiO_2_) clearly confirmed the binding of CdS@MPA QDs homogeneously distributed on the TiO_2_ surface with a large cargo. However, as shown in Figure S5 for CdS@MSA-TiO_2_, the number of CdS QDs on the TiO_2_ surface was lower than in the case of CdS@MPA QDs even when a high percentage of CdS QDs (50%) was used.

Figure S6 shows the XRD pattern of pristine TiO_2_ and CdS@MSA-TiO_2_ and CdS@MPA-TiO_2_ nanohybrids. The diffraction peaks centered at 25.4°, 37.9°, 48.1°, and 54.0° indexed to (101), (004), (200), and (105) crystalline planes of TiO_2_, respectively (anatase, JCPDS Card 21-1272).^64^ The peaks located at 27.5°, 36.2°, and 54.0° correspond to the (110), (101), and (211) planes of the rutile phase, respectively (JCPDS Card 21-1276), which are present in the commercial Degussa TiO_2_,^67^ while the peaks at 26.6° and 44.0° confirm the successful attachment of CdS QDs to the TiO_2_ surface in the nanohybrids and are consistent with the analyses of HRTEM images discussed above.

The ATR-FTIR (attenuated total reflectance-Fourier transform infrared) spectra for both hybrids, the CdS QDs, and their organic ligands (MPA and MSA) were recorded. The presence of CdS QDs and TiO_2_ nanoparticles was clearly distinguished in the nanohybrid (Figure S7). The intense stretching C=O bands for the MSA and MPA were identified at 1692 and 1705 cm^–1^, respectively. Nevertheless, the asymmetric and symmetric vibration bands of the carboxylate group were clearly observed below 1560 cm^–1^ for the mercapto propionic and mercapto succinic-capped CdS QDs and the nanohybrids. This finding agrees with data reported for other chalcogenide-capped QDs with mercapto alkyl carboxylic acids.^68,69^ The broad band observed below 800 cm^–1^ corresponds to the Ti–O stretching characteristic band of the TiO_2_.^70^

The diffuse reflectance spectra of the CdS@MPA-TiO_2_ hybrid showed the presence of both components, TiO_2_ and CdS@MPA nanoparticles (Figure S8). Applying the Kubelka–Munk [K–M or *F*(*R*)] method,^71^ a band gap of 3.03 eV for TiO_2_ in the hybrid was extracted from the slope of the plots of [*F*(*R*) × *E*]^1/2^ versus photon energy (*E*, electronvolts) in Figure S8. This value was lower than the value of 3.26 eV obtained for pristine TiO_2_ (reported value of 3.2 eV for Degussa TiO_2_ P25 nanoparticles),^72^ while the band gap of CdS@MPA was maintained at 2.25 eV. A similar red shift was observed for the TiO_2_ nanofibers coated with CdS.^22^ Furthermore, this finding confirms the synergy between the nanoparticles in the hybrids due to the efficient binding of CdS@MPA to the TiO_2_ surface, which was supported by the different techniques discussed above.

### Oxidative Hydroxylation of Aryl Boronic Acids with Pristine CdS QDs as a Photocatalyst

The photocatalytic activity of pristine CdS QDs was evaluated in the hydroxylation of aryl boronic acids under visible light irradiation in aqueous media (Table S1). The irradiation of a phenylboronic acid (**1a**) solution in the presence of CdS@MPA or CdS@MSA at 10 wt % and triethylamine (TEA, 5.0 equiv), as the sacrificial electron donor, was carried out in a Rayonet photoreactor (eight visible lamps, emission centered at 420 nm) in a saturated oxygen atmosphere for 18 h to produce phenol (**2a**) with chemical yields of 75% (CdS@MPA) and 77% (CdS@MSA). Control experiments carried out using CdS@MPA QDs as the photocatalyst revealed that the presence of amine as the electron donor, oxygen as the oxidant, and visible light was essential for the photocatalytic formation of phenol **2a**.

Compared with conventional irradiation methods, light-emitting diode lamps (LED lamps) have a lower power consumption and voltage, a longer lifetime, a smaller size, minimal heat generation, and narrower emission spectra, which allows a more selective photocatalyst irradiation.^73,74^ Therefore, the reaction was conducted using blue LED (3 W, λ_max_ = 467 nm), and the optimal conditions were further explored (Table S2). The photocatalyst loading was screened at 5 and 10 wt % (Table S2, entries 1 and 2), producing **2a** with yields of 24% and 33%, respectively, after irradiation for 12 h. Under an air atmosphere as the oxygen source, **2a** was obtained with an only 3% yield (Table S2, entry 3), thus confirming the essential role of an oxygen-saturated atmosphere for the photocatalytic transformation. Then, different numbers of equivalents of TEA were tested (Table S2, entries 4–6); **2a** was obtained with a yield of 55% when using 5 equiv of TEA, after irradiation for 16 h (Table S2, entry 4), and the yield increased to 89% after irradiation for 24 h (Table S2, entry 7). To expand its use in organic synthesis, the photocatalytic reaction was also carried out in acetonitrile (MeCN), which exhibits low toxicity and in which oxygen is more soluble than in water.^75,76^ Nevertheless, **2a** was not produced in acetonitrile (Table S2, entry 8) due to the poor dispersibility of CdS@MPA in this solvent.

To explore the synthetic scope for the oxidative hydroxylation of arylboronic acids, a wide range of arylboronic acids were efficiently hydroxylated to the corresponding aryl alcohols in moderate to good yields (41–89%) under the optimized reaction conditions indicated above. As shown in Table S3, arylboronic acids (**1a**–**e**) with electron-donating and -withdrawing groups were effectively converted into the desired aryl alcohols (**2a**–**e**, respectively).

*Ortho*-substituted aryl boronic acid substrates with methyl (**1b**), methoxy (**1c**), and chloride (**1e**) substituents gave aryl alcohols **2b**, **2c**, and **2e** in 76%, 41%, and 76% yields, respectively. In addition, the substrate with a methoxy group at the *para* position (**1d**) yielded aryl alcohol **2d** in 59% yield. These results indicate that the electronic effects or steric hindrance of the substituent do not affect product formation.

### Oxidative Hydroxylation of Aryl Boronic Acids and Esters Using the CdS@MPA-TiO_2_ Nanohybrid as the Photocatalyst

The selective excitation of CdS@MPA QDs in the CdS@MPA-TiO_2_ nanohybrid was easily achieved by using blue LED lamps centered at 467 nm, taking into account the band gap of each semiconductor.^77^ The hydroxylation of **1a** was carried out under the optimized conditions (6.7 mg of CdS@MPA-TiO_2_ or 1.7 mg of CdS@MPA, 5 mg of TiO_2_, 5 equiv of TEA, saturated O_2_ atmosphere, 5.0 mL of H_2_O, irradiation with a 3 W blue LED centered at 467 nm), and the results were compared with those obtained with the pristine CdS semiconductor. Product **2a** was obtained in an 81% yield when using the nanohybrid, compared with a 41% yield for CdS@MPA QDs after 12.5 h of irradiation. Control experiments showed a significant decrease in the reaction yield to 35% when a mechanical mixture of CdS@MPA and TiO_2_ was used, while pristine TiO_2_ led to an only 6% yield of the product. These results evidenced that the close contact between both nanoparticles in the nanohybrid, mediated by the MPA ligand, favors the charge transfer between them and reduces the band gap of the TiO_2_ in the nanohybrid [diffuse reflectance measurements (Figure S8)], thereby improving the photocatalytic response.

A wide array of aryl boronic acids/esters was also tested to explore the potential of the CdS@MPA-TiO_2_ nanohybrid as a photocatalyst. Thus, aryl boronic acids with different substituents were efficiently converted into the corresponding phenols with good to excellent yields [56–100% (Table 1)] after 24 h of irradiation.

**Table 1:**
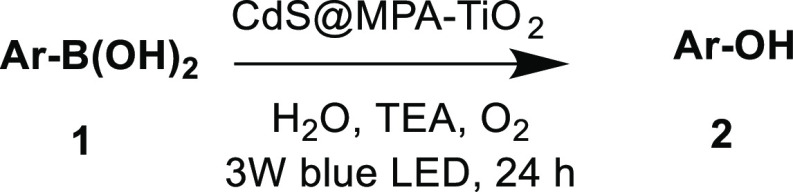
Scope of the Oxidative Hydroxylation of Aryl Boronic Acids Using CdS@MPA-TiO_2_ as the Photocatalyst^a^

entry	substrate	Ar	chemical yield of product (**2**) (%)
1	**1a**	C_6_H_5_	100 (86)^b^
2	**1b**	2-CH_3_C_6_H_4_	94 (90)^b^
3	**1c**	2-CH_3_OC_6_H_4_	56
4	**1d**	4-CH_3_OC_6_H_4_	77 (75)^b^
5	**1e**	2-ClC_6_H_4_	92 (90)^b^
6	**1f**	2,6-(CH_3_O)_2_C_6_H_3_	35
7	**1g**	2,4,6-(CH_3_)_3_-C_6_H_2_	63
8	**1h**	4-NO_2_C_6_H_4_	0
9	**1i**	4-pyridyl	0
10	**1j**	4-CO_2_H-C_6_H_4_	89 (85)^b^

a Reaction conditions: **1** (0.1 mmol), CdS@MPA-TiO_2_ (10 wt %), TEA (5 equiv), O_2_ (saturated atmosphere), H_2_O (5.0 mL), 3 W blue LED (467 nm), 24 h. All of the yields were calculated by ^1^H NMR using sodium terephthalate as an internal standard (see Figures S9–S16).

b Chemical yield obtained from the isolated products (see Figures S9, S10, S12, S13, and S16).

While the yield of phenol and *o*-cresol was excellent for aryl boronic acids **1a** and **1b**, the chemical yield decreased to 56% and 77% for boronic acids with a methoxy group at the *ortho* (**1c**) and *para* positions (**1d**), respectively, and it was even lower for 2,6-dimethoxyphenylboronic acid (**1f**), for which the corresponding phenol was obtained with a moderate chemical yield (35%), thus suggesting that both steric and electronic effects play a role in the effectiveness of the reaction (Table 1, entries 1–4 and 6). Similarly, 2,4,6-trimethylphenylboronic acid (**1g**) afforded **2g** in a 63% yield, compared with a 94% yield for **2b** with the methyl group at the *ortho* position (Table 1, entries 7 and 2, respectively). Moreover, the strong electron-withdrawing nitro substituent in **1h**, as well as the electron-poor ring of **1i**, failed to produce the corresponding phenol. Pyridinyl boronic acids have been converted into the corresponding phenols in good chemical yields by hydroxylation with *N*-oxides.^26^ However, substrates bearing strong electron-withdrawing substituents, such as 4-pyridyl and 4-nitrophenyl, could compete with the oxygen for the electron in the TiO_2_ CB, thus decreasing the extent of formation of the superoxide radical anion necessary to carry out the oxidative hydroxylation.

It is noteworthy that 4-carboxyphenylboronic acid (**1j**) produced the corresponding carboxyphenol **2j** in an 89% yield. The presence of TEA regulated the pH to favor the presence of the carboxylate anion, which is considered an electron donor substituent.^78^

**Scheme 2:**
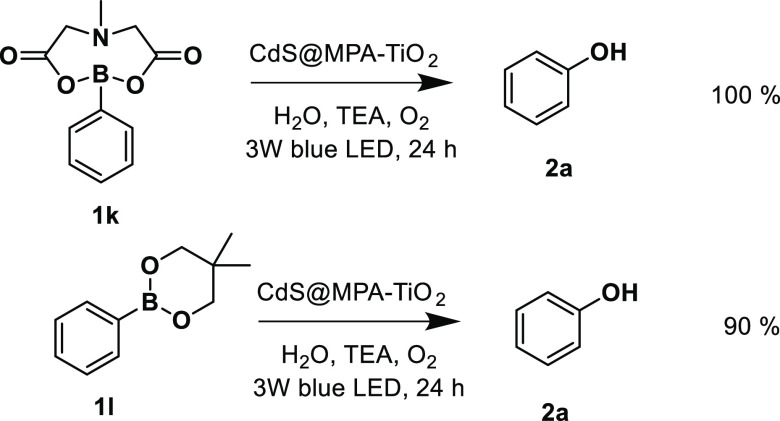
Extended Scope of the Reaction to Produce Phenyl Boronic Esters **1k** and **1l** The chemical yield was calculated by ^1^H NMR spectroscopy using the relative area method (sodium terephthalate was added as the internal standard).

This protocol is not restricted to aryl boronic acids, but it can be applied to the oxidative hydroxylation of aryl boronic esters (Scheme 2). To this end, phenylboronic acid *N*-methyliminodiacetic ester (**1k**) and phenylboronic acid neopentylglycol ester (**1l**) were synthesized according to reported protocols and the products were calculated by NMR spectroscopy (see the Supporting Information for further details and Figures S17 and S18).^79−81^

The oxidative hydroxylation of phenylboronic acid *N*-methyliminodiacetic ester (**1k**) and phenylboronic acid neopentylglycol ester (**1l**) using the CdS@MPA-TiO_2_ nanohybrid produced phenol **2a** in 100% and 90% yields, respectively.

**Scheme 3:**
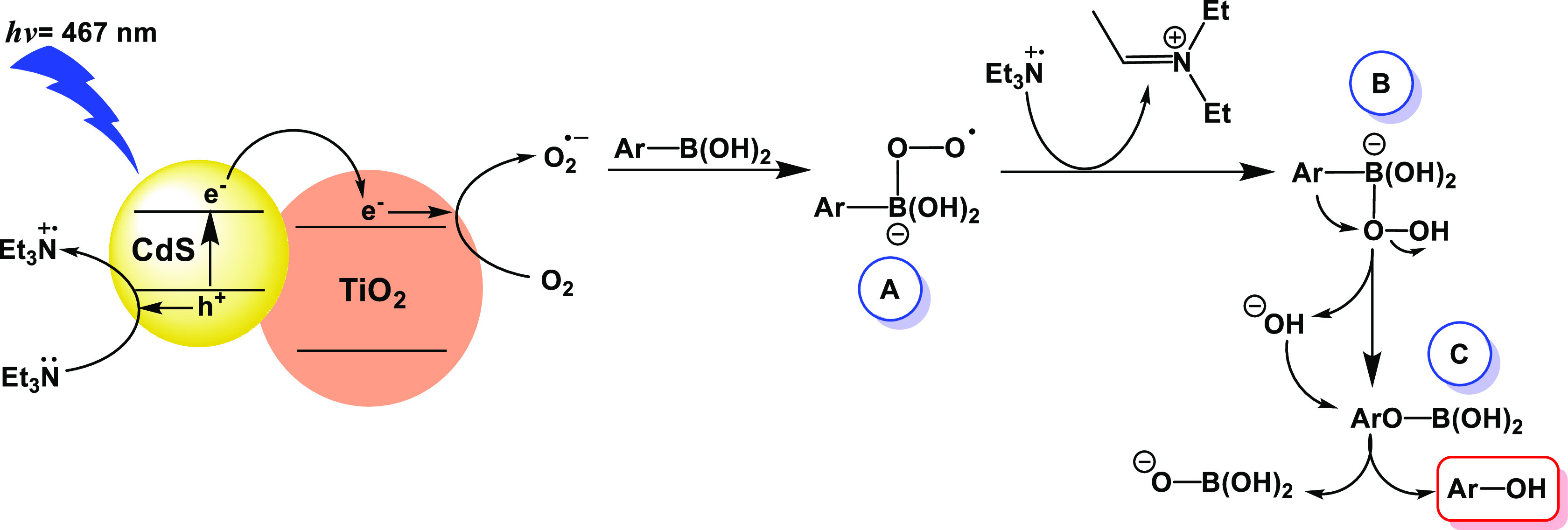
Proposed Mechanism for the Oxidative Hydroxylation of Aryl Boronic Acids/Esters Using the CdS@MPA-TiO_2_ Hybrid as a Photocatalyst

On the basis of the results described above, and previously reported,^42,44^ the mechanism proposed for the oxidative hydroxylation of aryl boronic acids is that depicted in Scheme 3. First, the excitation at 467 nm of CdS in the CdS@MPA-TiO_2_ hybrid affords exciton formation (electron–hole pair) followed by an electron migration from the CB of CdS to the CB of TiO_2_ due to their suitable band gap positions (Scheme 1). Then, single-electron transfer (SET) from the sacrificial electron donor TEA to the valence band of CdS produces the radical cation TEA^^•^+^, while oxygen reduction takes place by electron transfer from the TiO_2_ CB to oxygen, thereby forming the superoxide radical anion (O_2_^•–^) as the reactive oxygen species. The photogenerated O_2_^•–^ can then react with the aryl boronic acid (**1**) to produce the intermediate peroxydihydroxy(aryl)borate (intermediate **A**), which gives rise to intermediate **B** by hydrogen atom abstraction from TEA^•^^+^, as previously reported.^42^ Finally, the formation of the phenol (**2**) takes place by the rearrangement of **B** into **C** followed by its hydrolysis to yield the corresponding phenol (Scheme 3).

**Scheme 4:**
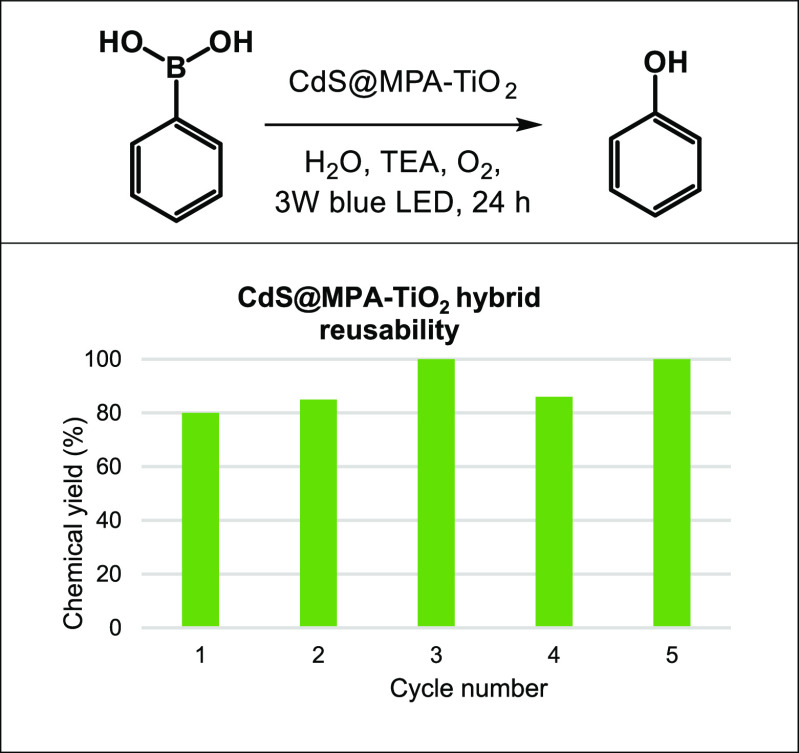
Reusability of CdS@MPA-TiO_2_ under the Optimized Reaction Conditions

Finally, the reusability of the CdS@MPA-TiO_2_ hybrid was analyzed using the oxidative hydroxylation of **1a** as a model reaction (Scheme 4). After irradiation for 24 h, the photocatalyst was recovered by centrifugation, given the heterogeneous nature of the photoreaction. Then, the photocatalyst was washed several times with ethanol and distilled water, dried under vacuum, and used for the next photocatalytic cycle. This test proved that the photocatalyst has excellent recycling capability because no significant loss of reactivity was observed up to five consecutive cycles, producing phenol **2a** in a high yield (90 ± 10%). Moreover, the photocatalyst integrity of the CdS@MPA-TiO_2_ was confirmed by HRTEM images and energy dispersive X-ray spectroscopy (EDS) mapping analysis, recorded before and after five photocatalytic cycles for the oxidative hydroxylation of **1a** (Figure S19). The size, morphology, and composition of the hybrid were preserved, thereby suggesting the good chemical and photochemical stability of the nanohybrid under the reaction conditions.

## Conclusions

In summary, CdS@MPA-TiO_2_ hybrids were successfully used as colloidal visible light photocatalysts for the oxidative hydroxylation of a broad family of aryl boronic acids/esters to obtain their corresponding phenols with chemical yields of ≤100% under environmentally friendly conditions. The strong attachment of the CdS to the TiO_2_ surface, mediated by the mercapto alkyl acids, ensured the close contact between the nanoparticles, thereby favoring the injection of electrons from the conduction band of CdS to TiO_2_ in the nanohybrid and enabling their reuse up to five catalytic cycles without a significant loss of activity. The higher photocatalytic efficiency of CdS@MPA-TiO_2_ hybrids, compared with that of pristine CdS nanoparticles and the mechanical mixture, resulted from the synergy between both nanoparticles, thus reducing the extent of charge recombination in the nanohybrid. The presence of oxygen as the oxidant was crucial for the phototransformation of aryl boronic acids in phenols, which agrees with the *in situ* generation of the superoxide radical anion (O_2_^•–^) under the photocatalytic conditions. Finally, this study illustrates how this approach can be applied to the preparation of a new class of heterogeneous photocatalysts of interest in the field of organic synthesis.

## Experimental Section

### Materials and Methods

Aryl boronic acids and titanium(IV) oxide Aeroxide P25 were obtained from Aldrich Chemical Co. Reagents and solvents were of the highest quality available and used without further purification. All products are known compounds and were characterized by comparison with published ^1^H NMR and ^13^C NMR data.^33,82^^1^H NMR spectra were recorded using D_2_O as a solvent. UV–vis absorption spectra were recorded on a UV–vis 1800 Shimadzu spectrometer. ^1^H and ^13^C NMR spectra were registered on a Bruker AC-400 (400 MHz) spectrometer, and all spectra were reported in δ (parts per million). Centrifugation was carried out using Eppendorf model 5804 centrifuges with a model F-34-6-38 rotor. HRTEM images were recorded with a TECNAI G2 F20 (FEI) electron microscope with a field emission transmission of 200 kV (FEG) with 0.24 nm resolution (point resolution). The reaction mixtures were irradiated in a homemade photoreactor equipped with a 3 W blue LED from Demasled (centered at 467 nm, luminous flux of 22 lm, and full width at half-maximum of 33 nm) in a glass reaction vial at room temperature with an oxygen atmosphere ∼1 cm from the source. ATR-FTIR spectroscopy was carried out in a Bruker Alpha II platinum-ATR instrument. The software used for the data treatment was OPUS 8.1. The diffuse reflectance measurements were carried out on a UV/vis/NIR Lambda 1050 spectrophotometer, equipped with the software PerkinElmer UV Winlab.

### Synthesis of CdS@MPA and CdS@MSA QDs

For the synthesis of CdS@MPA QDs, 120 mL of a water solution of CdCl_2_ (0.02 M) was added to a 250 mL round-bottom flask. Then, mercaptopropionic acid (MPA, 0.014 mol) was introduced under mild stirring conditions. The pH of the solution was adjusted to 11 using 1.0 M NaOH. Thereafter, H_2_O_2_ [300 μL of a 30% (w/w) solution] was added dropwise to the solution, and the reaction mixture was refluxed for 1, 2, and 24 h in a hot plate. The formed QD nanocrystals were centrifuged and washed thoroughly with ethanol to remove unreacted reagents. Finally, these QD nanocrystals were vacuum-dried to afford a yellow solid.

For the synthesis of CdS@MSA QDs, 120 mL of a water solution of CdCl_2_ (0.02 M) was added to a 250 mL round-bottom flask. Then, mercaptosuccinic acid (MSA, 0.014 mol) was introduced under mild stirring conditions. The pH of the solution was adjusted to 10 using 1.0 M NaOH. Thereafter, H_2_O_2_ [300 μL of a 30% (w/w) solution] was added dropwise to the solution, and the reaction mixture was refluxed for 1, 2, and 24 h. The formed QD nanocrystals were centrifuged and washed thoroughly with ethanol to remove unreacted reagents. Finally, these QD nanocrystals were vacuum-dried to afford a yellow solid.

### Synthesis of the CdS-TiO_2_ Nanohybrid

Briefly, TiO_2_ (67.5 mg), CdS@MSA, or CdS@MPA QDs (22.5 mg) were added to a 100 mL round-bottom flask with 45 mL of Milli-Q water. Then, 1.0 M NaOH (0.6 mL) and additional organic ligand MPA (1 mL, 15.0 mmol) or MSA (0.35 g, 2.3 mmol) were added. The resulting mixture was sonicated in an ultrasound bath at 50 °C for 1 h. Then, the sample was centrifuged at 10 °C for 15 min, and the pellet was dispersed in H_2_O (5 mL), followed by the addition of ethanol (40 mL) and centrifugation for 15 min (Figure S4). The isolated CdS-TiO_2_ hybrid was dried under vacuum for further characterization and use. CdS@MPA-TiO_2_ and CdS@MSA-TiO_2_ were obtained as a light yellow solid and an oily solid, respectively. The reaction was scaled up to 4 times for the scope of the reaction study.

### General Procedure for the Oxidative Hydroxylation of Aryl Boronic Acids/Esters

The reaction was carried out in a vial, using 1 mmol of aryl boronic acid or aryl boronic ester (1.0 equiv), CdS@MPA or CdS@MSA QDs (10 wt %), triethylamine (TEA, 5 equiv), and 5 mL of water under magnetic stirring. The vial was sealed, and the reaction mixture was saturated with oxygen by O_2_ bubbling for 10 min and then irradiated with 3 W blue LEDs for 24 h. Then, sodium terephthalate was added as an internal standard, and the mixture was stirred and then centrifuged at 2000 rpm. Then D_2_O was added to the crude mixture for ^1^H NMR quantification.

The product isolation procedure for the representative examples listed in Table 1 was carried out as follows. The reaction mixture was centrifuged at 2000 rpm, and the resulting supernatant was neutralized with HCl (0.1 M) up to pH 6–7 and extracted with ethyl acetate (10 mL) three times, dried with Na_2_SO_4_ anhydrous, and filtered. The solvent was removed under reduced pressure, and the compound was purified by flash chromatography.

### General Procedure for the Reusability of Nanohybrid CdS@MPA-TiO_2_ in the Oxidative Hydroxylation of Phenylboronic Acid (**1a**)

A reaction vial equipped with a magnetic stirring bar was charged with 0.1 mmol of phenylboronic acid, nanohybrid CdS@MPA-TiO_2_ (10 wt %), triethylamine (TEA, 5 mmol), and 5 mL of water. The vial was sealed, and the reaction mixture was saturated with oxygen by O_2_ bubbling for 10 min and irradiated with 3 W blue LEDs for 24 h. Then, the reaction mixture was centrifuged, the supernatant removed, sodium terephthalate (0.025 mmol) added as an internal standard, the supernatant mixture stirred, and an aliquot of the reaction mixture (250 μL) diluted with 250 μL of D_2_O for ^1^H NMR determination of the reaction yield.

The recovered QDs were washed several times with ethanol and vacuum-dried. Afterward, the QDs were used in a new photocatalytic reaction.

## Data Availability

The data underlying this study are available in the published article and its Supporting Information.

## Supporting Information Available

Additional spectra; results using pristine CdS QDs; microscopy and spectroscopy characterization of the hybrid; synthesis of phenylboronic acid *N*-methyliminodiacetic ester and phenylboronic acid neopentylglycol ester; and NMR spectra of synthesized phenylboronic esters and representative products listed in Table 1 (PDF)
