# Supplementary material for: Linker-Assisted CdS-TiO2 Nanohybrids as Reusable Visible Light Photocatalysts for the Oxidative Hydroxylation of Arylboronic Acids
Source: J Org Chem. 2023 Mar 17;88(10):6489–97. doi: 10.1021/acs.joc.2c02964 (PMC10204062; doi:10.1021/acs.joc.2c02964)
Supplement: Supplementary file 1 — jo2c02964_si_001.pdf [file jo2c02964_si_001.pdf]

Supporting Information

Linker-assisted CdS-TiO_2 nanohybrids as reusable visible-light photocatalysts for the oxidative hydroxylation of arylboronic acids

Willber D. Castro-Godoy,[a], [b] Luciana C. Schmidt,[a], [c] Diego Flores-Oña, [d], [e] Julia Pérez-Prieto,[d]* Raquel E. Galian[d]* and Juan E. Argüello[a]*

[a] INFIQC-CONICET-UNC, Dpto. de Química Orgánica, Facultad de Ciencias Químicas, Universidad Nacional de Córdoba, Ciudad Universitaria, X5000HUA. Córdoba, Argentina.
E-mail: jea@fcq.unc.edu.ar
[b] CENSALUD-UES, Departamento de Química, Física y Matemática, Facultad de Química y Farmacia, Universidad de El Salvador, Final Av. Mártires y Héroes del 30 de Julio, San Salvador, 1101, El Salvador.
[c] Instituto de Tecnología Química, Universitat Politècnica de València-Consejo Superior de Investigaciones Científicas, Avenida de los Naranjos s/n, 46022 Valencia, Spain.
[d] Institute of Molecular Science (ICMol), University of Valencia, Catedrático José Beltrán 2, Paterna, 46980 Valencia, Spain.
E-mails: raquel.galian@uv.es, julia.perez@uv.es
[e] Facultad de Ingeniería Química Universidad Central de Ecuador, Rither y Bolivia, Ciudadela Universitaria, 170521, Quito, Ecuador.

Table of contents

Figure S1: UV-visible absorption and emission spectra of CdS QDs (0.24 mg/ mL) water dispersion: (a, c) CdS@MSA QDs and (b, d) CdS@MPA QDs recorded at different reaction times: 1 (black), 2 (red) and 24 h (blue). Inset: photograph of the QDs dispersion at different reaction times. S4

Figure S2: HRTEM images of pristine CdS QDs: (a, b) CdS@MSA and (c, d) CdS@MPA. Scale bar 20 nm (a, c) and 2 nm (b, d). S5

Figure S3: XRD spectra of (a) CdS@MSA and (b) CdS@MPA. References for the CdS cubic phase (red line) and CdS hexagonal phase (green line), taken from COD (Crystallography Open Database), cubic CdS file No. 96-900-0068 and hexagonal CdS file No. 96-900-8863. .... S6

Figure S4: Images of (a) water dispersion of CdS@MSA (1) and CdS@MPA (2), (b) mixture of CdS QDs (20 %) and TiO_2, and (c) isolated nanohybrid CdS@TiO_2 after centrifugations steps for both QDs. S7

Figure S5: HRTEM images of CdS@MSA-TiO_2 hybrid. Scale bar 20 nm. S8

Figure S6: XRD spectra of anatase TiO_2 (black) and the nanohybrid CdS@MPA-TiO_2 (green line) and CdS@MSA-TiO_2 (dark red line). *Peaks corresponding to rutile phase of TiO_2. Orange lines correspond to the main crystal planes for cubic CdS. S9

Figure S7: ATR-FTIR spectra of the nanohybrids, CdS QDs and their corresponding organic capping: a) CdS@MSA-TiO_2 (dark red line) CdS@MSA (blue) and MPA (black line); and b) CdS@MPA-TiO_2 (green line), CdS@MPA (blue line) and MPA (black line). S10

Table S1: Oxidative hydroxylation of arylboronic acids with pristine CdS QDs s photocatalyst [a] S11

Table S2: optimization of the photocatalytic conditions for phenylboronic acid oxidative hydroxylation using CdS@MPA as photocatalyst. [a] S12

Table S3: Oxidative hydroxylation of arylboronic acids using pristine CdS@MPA as photocatalyst. [a] S13

Figure S8: Diffuse reflectance spectra and their corresponding $[F(R)*E]^{1/2}$ vs. E for the CdS@MPA-TiO_2 hybrid (●), CdS@MPA QDs (■) and TiO_2 nanoparticles (▲). S14

Figure S9: Phenol (2a). Isolated by column chromatography on silica gel using a mixture of ethyl acetate and pentane (1:9) as mobile phase to afford red solid in 86 % yield (8.1 mg). ^{1}H-NMR (400 MHz, $CDCl_3$): δ= 7.23 (t, *J*= 8 Hz, 2H), 6.93 (t, *J*= 7.4 Hz, 1H), 6.83 (d, *J*= 8 Hz, 2H). ^{13}C{^{1}H} -NMR (101 MHz, $CDCl_3$): δ= 155.5, 129.8, 121, 115.5.[1] S15

Figure S10: *o*-cresol (2b). Isolated by column chromatography on silica gel using a mixture of ethyl acetate and pentane (1:9) as mobile phase to afford colorless solid in a 90 % yield (9.7 mg). ^{1}H-NMR (400 MHz, $CDCl_3$): δ= 7.14 (d, J = 8 Hz, 1H), 7.10 (t, J = 7.8 Hz, 1H), 6.86 (t, J = 7.4 Hz, 1H), 6.78 (d, J = 8 Hz, 1H), 2.27 (s, 3H). ^{13}C{^{1}H}-NMR (101 MHz, $CDCl_3$): δ= 153.9, 131.2, 127.3, 123.9, 120.9, 115.0, 15.8.[2] S16

Figure S11: *o*-methoxyphenol (2c). quantified by ^{1}H-NMR and compared with an authentic sample. ^{1}H-NMR (400 MHz, $CDCl_3$): δ= 6.96–6.93 (m, 1H), 6.91–6.86 (m, 3H), 3.89 (s, 3H). ^{13}C{^{1}H}-NMR (101 MHz, $CDCl_3$): δ= 146.7, 145.9, 121.6, 120.3, 114.7, 110.9, 56.[3] S17

Figure S12: *p*-methoxyphenol (2d). Isolated by column chromatography on silica gel using a mixture of ethyl acetate and pentane (1:9) as mobile phase to afford white solid in a 75 % yield (9.3 mg).^{1}H-NMR (400 MHz,

$CDCl_3$): δ= 6.79–6.78 (m, 4H), 4.82 (s, 1H), 3.77 (s, 3H). $^{13}C\{^1H\}$-NMR (101 MHz, $CDCl_3$): δ 153.9, 149.7, 116.2, 115.1, 56.[1] S18

Figure S13: *o*-chlorophenol (2e). Isolated by column chromatography on silica gel using a mixture of ethyl acetate and pentane (1:9) as mobile phase to afford colorless oil in a 90 % yield (11.5 mg). ^1H-NMR (400 MHz, $CDCl_3$): δ= 7.31 (d, *J*= 7.8 Hz, 1H), 7.18 (t, J=7.5 Hz, 1H), 7.02 (d, *J*= 8 Hz, 1H), 6.87 (t, J = 7.8 Hz, 1H). $^{13}C\{^1H\}$-NMR (101 MHz, $CDCl_3$): δ= 151.6, 129.2, 128.6, 121.5, 120.1, 116.4.[2] S19

Figure S14: 2,6-dimethoxyphenol (2f). quantified by ^1H-NMR and compared with an authentic sample. ^1H-NMR (400 MHz, $CDCl_3$): δ= 6.80 (t, *J*= 8.4 Hz, 1H), 6.58 (d, *J*= 8.5 Hz, 2H), 3.88 (s, 6H). $^{13}C\{^1H\}$-NMR (101 MHz, $CDCl_3$): δ= 147.4, 135.0, 119.2, 105.1, 56.4.[1] S20

Figure S15: 2,4,6-trimethylphenol (2g). quantified by ^1H-NMR and compared with an authentic sample. ^1H-NMR (400 MHz, $CDCl_3$): δ= 6.79 (s, 2H), 2.21 (s, 9H). $^{13}C\{^1H\}$-NMR (101 MHz, $CDCl_3$): δ= 150.0, 129.4, 129.3, 122.9, 20.5, 15.9.[3] S21

Figure S16: *p*-hydroxybenzoic acid (2j). Isolated by column chromatography on silica gel using a mixture of ethyl acetate and pentane (1:1) as mobile phase to afford white solid in 85 % yield (11.7 mg).^1H-NMR (400 MHz, DMSO-d_6): δ= 7.78 (d, J = 8.6 Hz, 2H), 6.82 (d, J = 8.6 Hz, 2H). $^{13}C\{^1H\}$-NMR (100 MHz, DMSO-d_6): δ= 167.2, 161.6, 131.5, 121.4, 115.1.[3] S22

Synthesis of phenylboronic acid MIDA ester (6-methyl-2-phenyl-1,3,6,2-dioxazaborocane-4,8-dione) S23

Synthesis of Phenylboronic acid neopentylglycol ester (5,5-dimethyl-2-phenyl-1,3,2-dioxaborinane) S23

Figure S17: 6-methyl-2-phenyl-1,3,6,2-dioxozaborocano-4,8-dione (1k). ^1H-NMR (400 MHz, $(CD_3)_2CO$): δ= 7.52-7.54 (m, 2H), 7.35-7.37 (m, 3H), 4.34 (d, *J*= 17 Hz, 2H), 4.14 (d, *J*= 17 Hz, 2H), 2.72 (s, 3H). $^{13}C\{^1H\}$-NMR (101 MHz, $(CD_3)_2CO$): δ= 169.4, 133.5, 130.0, 128.7, 62.9, 48.4.[4, 5] S24

Figure S18: 5,5-dimethyl-2-phenyl-1,3,2-dioxoborinane (1l). ^1H-NMR (400 MHz, $CDCl_3$): δ= 7.80 (d, *J*=7 Hz, 2H), 7.43 (t, *J*= 7.2 Hz, 1H), 7.35 (t, J = 7 Hz, 2H), 3.78 (s, 4H), 1.03 (s, 6H). $^{13}C\{^1H\}$-NMR (101 MHz, $CDCl_3$): δ= 134.0, 130.8, 127.7, 72.5, 32.0, 22.1.[6] S25

Figure S19: HRTEM images of the CdS@MPA-TiO2 hybrid a) before and after b) 5 photocatalytic cycles for the oxidative hydroxylation of 1a (scale bar = 200 and 100 nm, respectively). Energy dispersive X-ray spectroscopy (EDS) mapping analysis of the CdS@MPA-TiO2 hybrid c) before and d) after 5 photocatalytic cycles for the oxidative hydroxylation of 1a. Elements detected and homogeneously distributed in the material: Cd (red), S (pink), Ti (green) and O (yellow). S26

References: S27

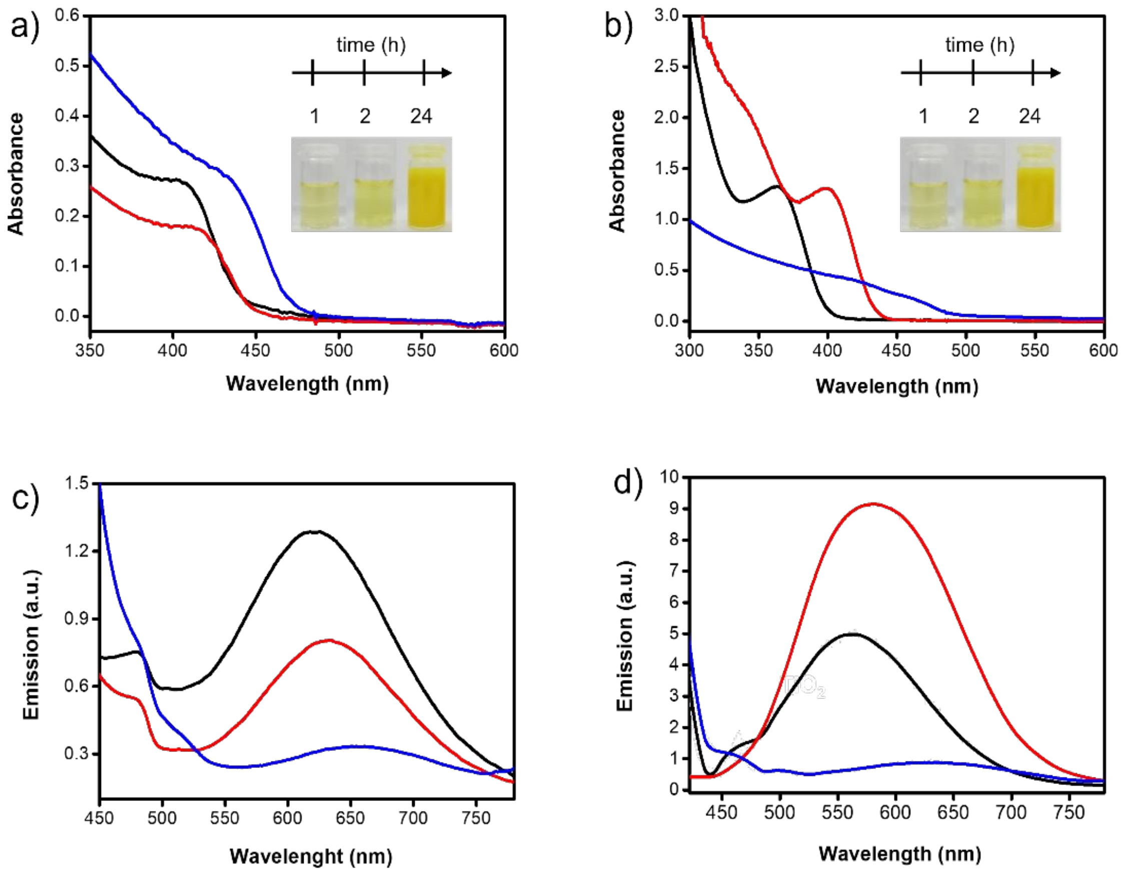

Figure S1: UV-visible absorption and emission spectra of CdS QDs (0.24 mg/ mL) water dispersion: (a, c) CdS@MSA QDs and (b, d) CdS@MPA QDs recorded at different reaction times: 1 (black), 2 (red) and 24 h (blue). Inset: photograph of the QDs dispersion at different reaction times.

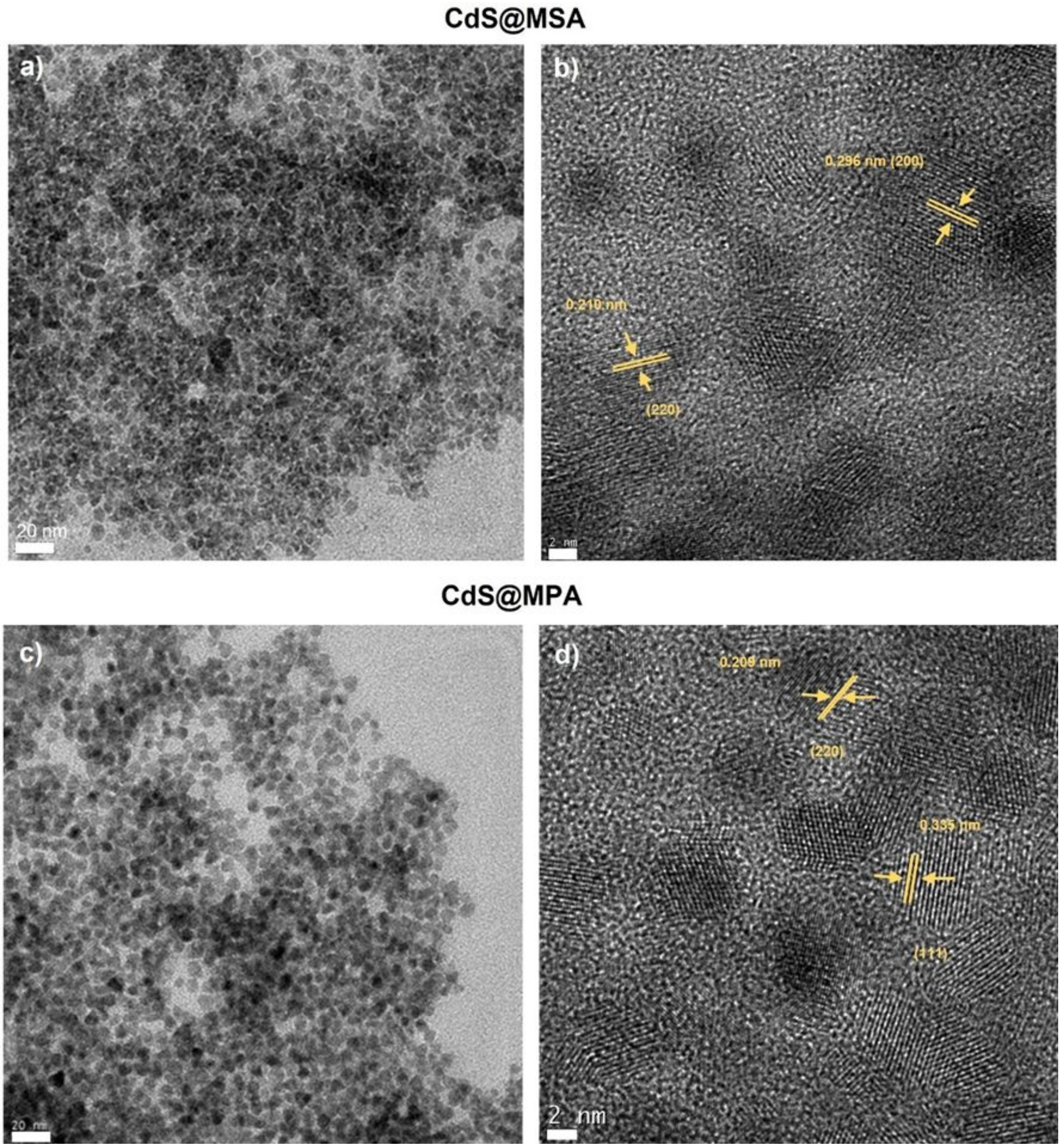

Figure S2: HRTEM images of pristine CdS QDs: (a, b) CdS@MSA and (c, d) CdS@MPA. Scale bar 20 nm (a, c) and 2 nm (b, d).

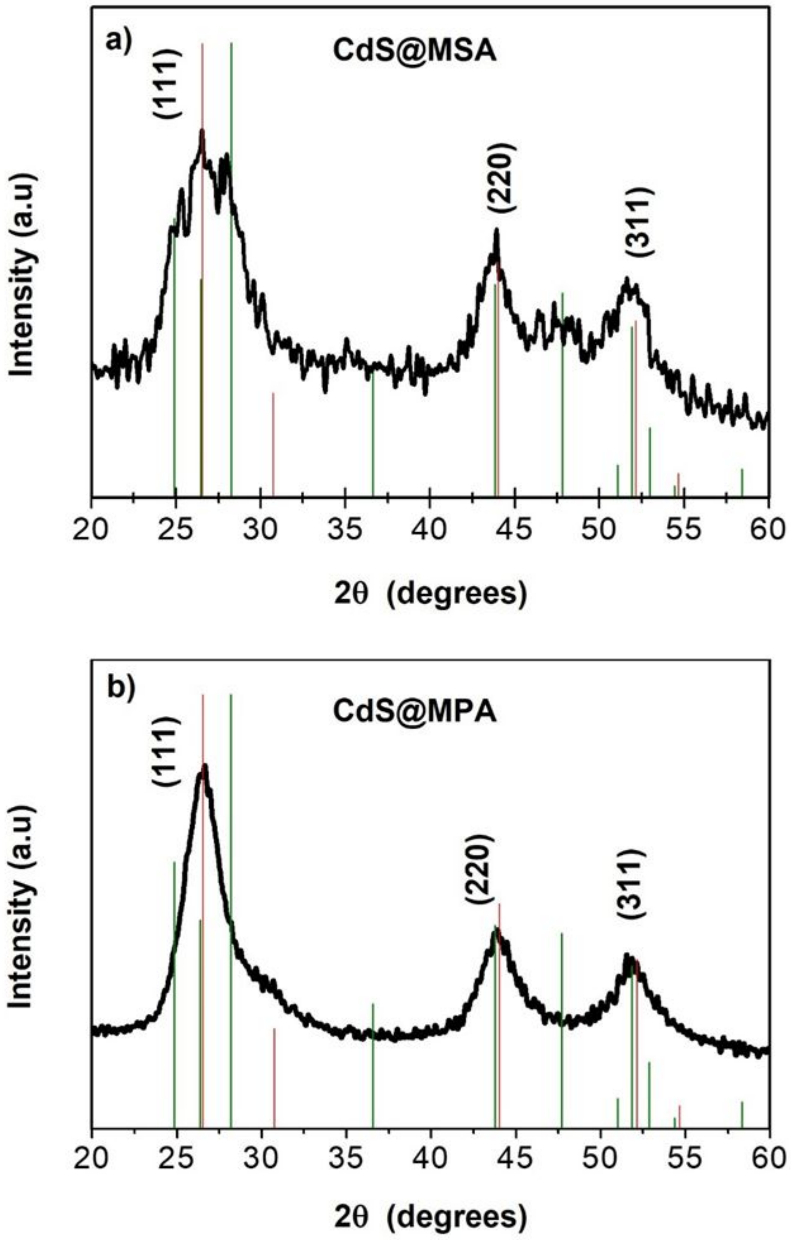

Figure S3: XRD spectra of (a) CdS@MSA and (b) CdS@MPA. References for the CdS cubic phase (red line) and CdS hexagonal phase (green line), taken from COD (Crystallography Open Database), cubic CdS file No. 96-900-0068 and hexagonal CdS file No. 96-900-8863.

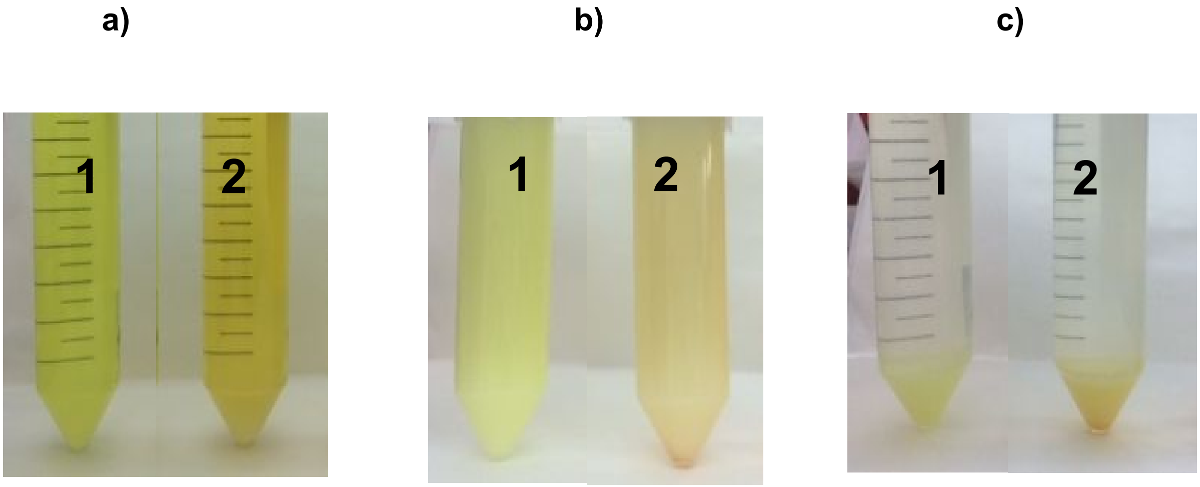

Figure S4: Images of (a) water dispersion of CdS@MSA (1) and CdS@MPA (2), (b) mixture of CdS QDs (20 %) and TiO_2, and (c) isolated nanohybrid CdS@TiO_2 after centrifugations steps for both QDs.

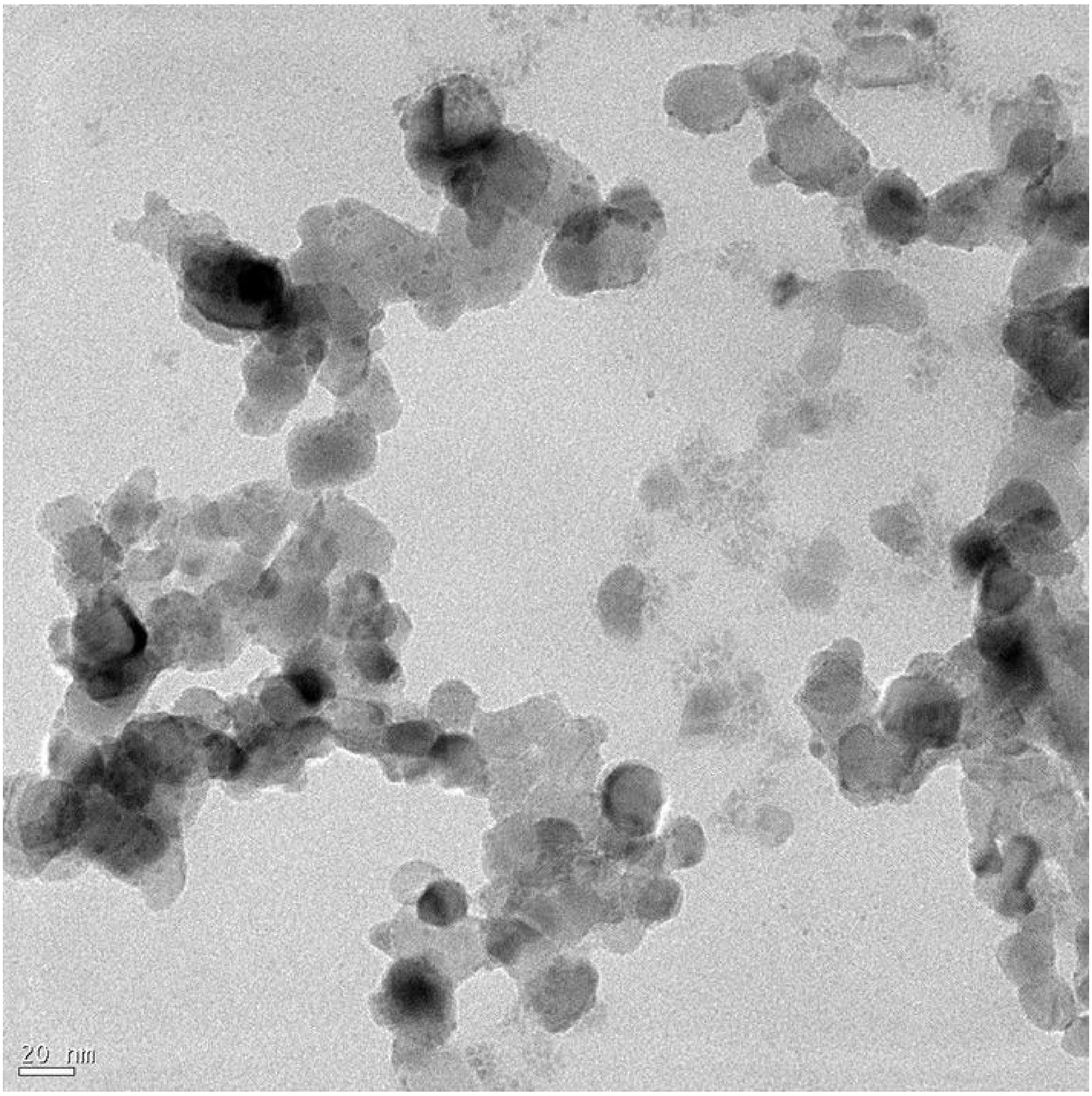

Figure S5: HRTEM images of CdS@MSA-TiO_2 hybrid. Scale bar 20 nm.

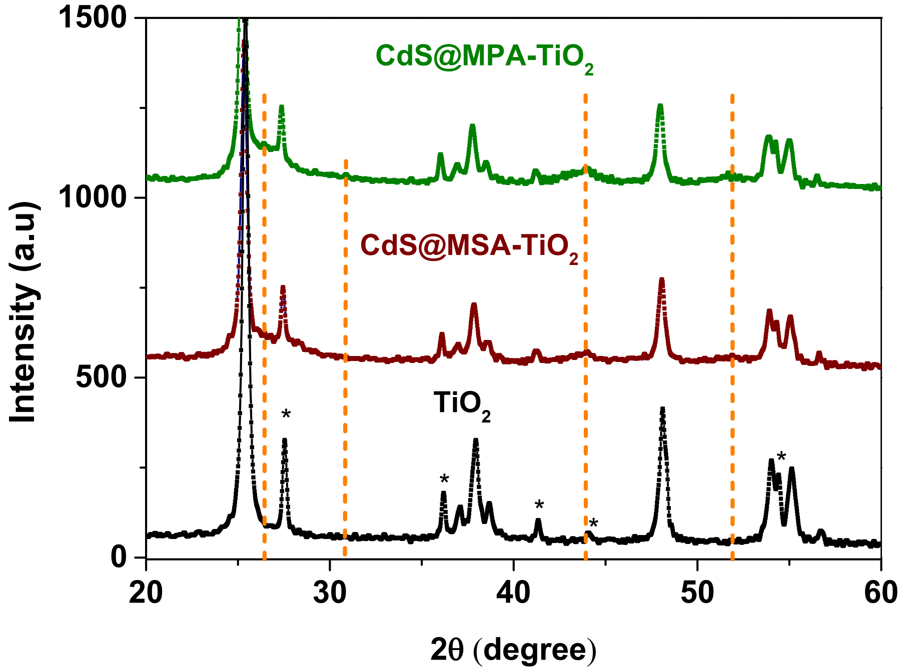

Figure S6: XRD spectra of anatase TiO_2 (black) and the nanohybrid CdS@MPA-TiO_2 (green line) and CdS@MSA-TiO_2 (dark red line). *Peaks corresponding to rutile phase of TiO_2. Orange lines correspond to the main crystal planes for cubic CdS.

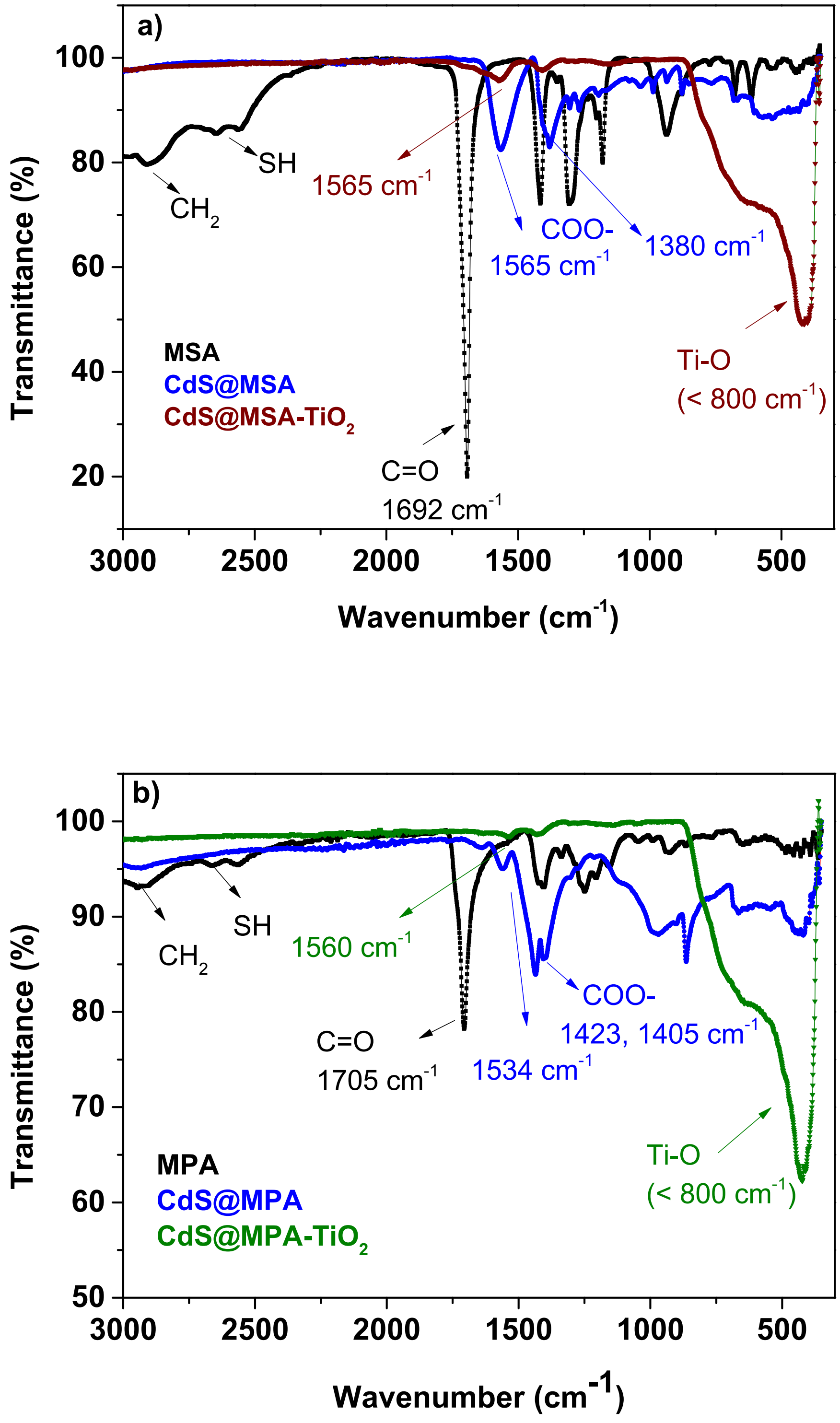

Figure S7: ATR-FTIR spectra of the nanohybrids, CdS QDs and their corresponding organic capping: a) CdS@MSA-TiO_2 (dark red line) CdS@MSA (blue) and MPA (black line); and b) CdS@MPA-TiO_2 (green line), CdS@MPA (blue line) and MPA (black line).

Table S1: Oxidative hydroxylation of arylboronic acids with pristine CdS QDs s photocatalyst [a]

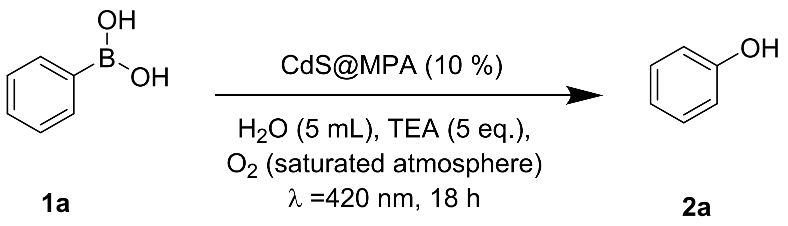

Entry	Lamps (centered at 420 nm)	Photocatalyst CdS@MPA	O_2	TEA	Product **2a** chemical yield (%)[b]
1	+	+	+	+	75
2	+	-	+	+	n.r.
3	-	+	+	+	n.r.
4	+	+	+	-	n.r.
5	+	+	-	+	n.r.

[a] Reaction conditions: **1a** (0.1 mmol), CdS@MPA (10 wt.%), TEA (5 equiv.), O_2 (saturated atmosphere), H_2O (5.0 mL), irradiation with 8 lamps centered at 420 nm, for 18 h. [b] Chemical yield obtained from the isolated product. n.r.= no reaction.

Table S2: optimization of the photocatalytic conditions for phenylboronic acid oxidative hydroxylation using CdS@MPA as photocatalyst. [a]

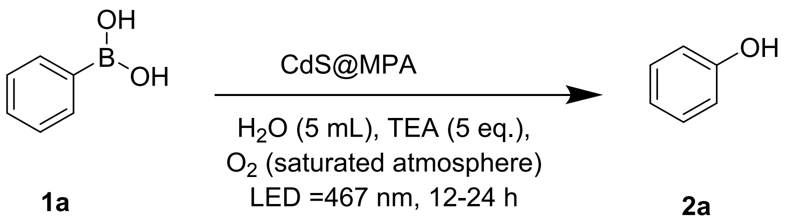

Entry	CdS@MPA (wt.%)	O_2 Source	TEA (equiv.)	Irradiation time (h)	Product **2a** chemical yield (%)[b]
1	5	saturated atmosphere	5	12	24
2	10	saturated atmosphere	5	12	33
3	10	air	5	12	3
4	10	saturated atmosphere	5	16	55
5	10	saturated atmosphere	3	16	38
6	10	saturated atmosphere	1	16	33
**7**	**10**	**saturated atmosphere**	**5**	**24**	**89**
8[c]	10	saturated atmosphere	5	24	n.r.

[a] Reaction conditions: **1a** (0.1 mmol.), CdS@MPA, TEA, O_2 source, solvent (5.0 mL), 3W blue LED (467 nm). [b] Yields were determined by ^{1}H-NMR using the relative areas method. [c] Acetonitrile was used as a solvent. n.r.= no reaction.

Table S3: Oxidative hydroxylation of arylboronic acids using pristine CdS@MPA as photocatalyst. [a]

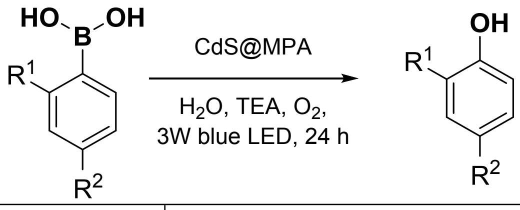

Substrate (1)	Product (2)	Chemical Yields (%)
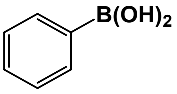 1a	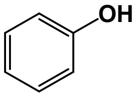 2a	89 [b]
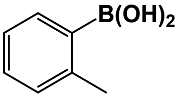 1b	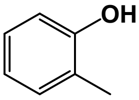 2b	76 [b]
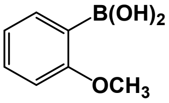 1c	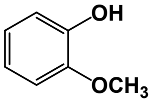 2c	41[b]
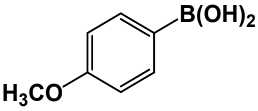 1d	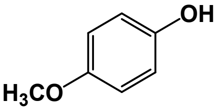 2d	59 [b]
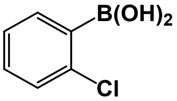 1e	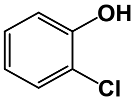 2e	76 [b]

[a] Reaction conditions: 1 (0.1 mmol), CdS@MPA (10 wt.%), TEA (5 equiv.), O_2 (saturated atmosphere), H_2O (5.0 mL), 3W blue LED (467 nm), 24 h. [b] Yield calculated by ^{1}H-NMR using the relative areas method.

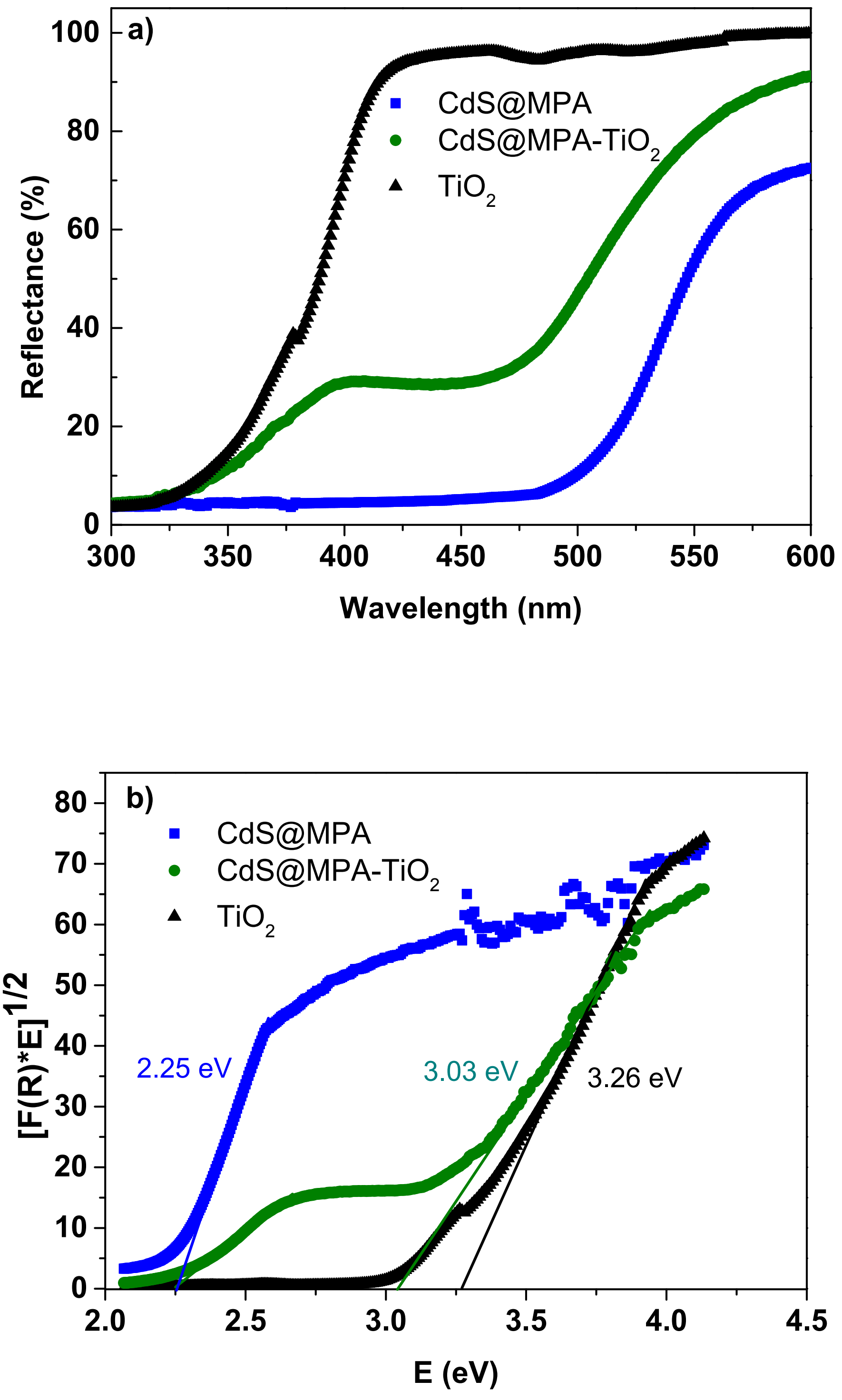

Figure S8: Diffuse reflectance spectra and their corresponding $[F(R)*E]^{1/2}$ vs. E for the CdS@MPA-TiO_2 hybrid (●), CdS@MPA QDs (■) and TiO_2 nanoparticles (▲).

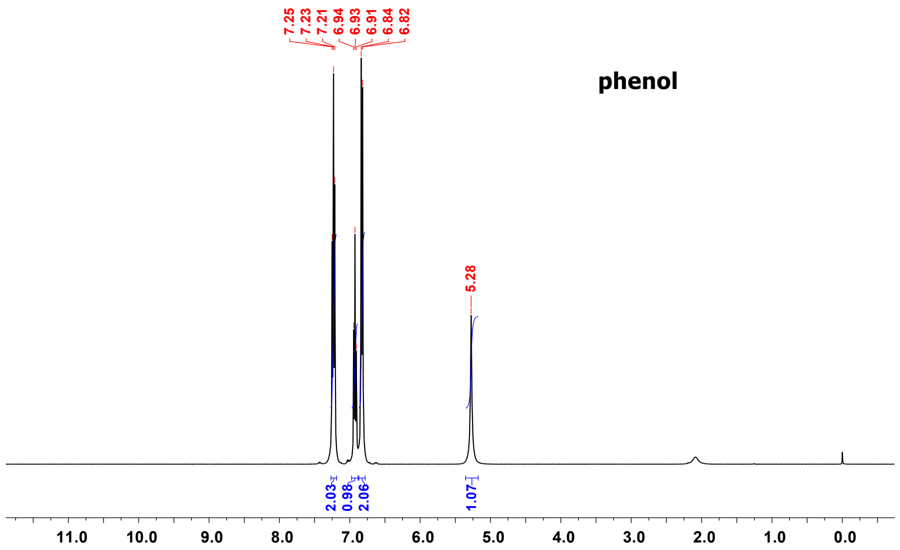

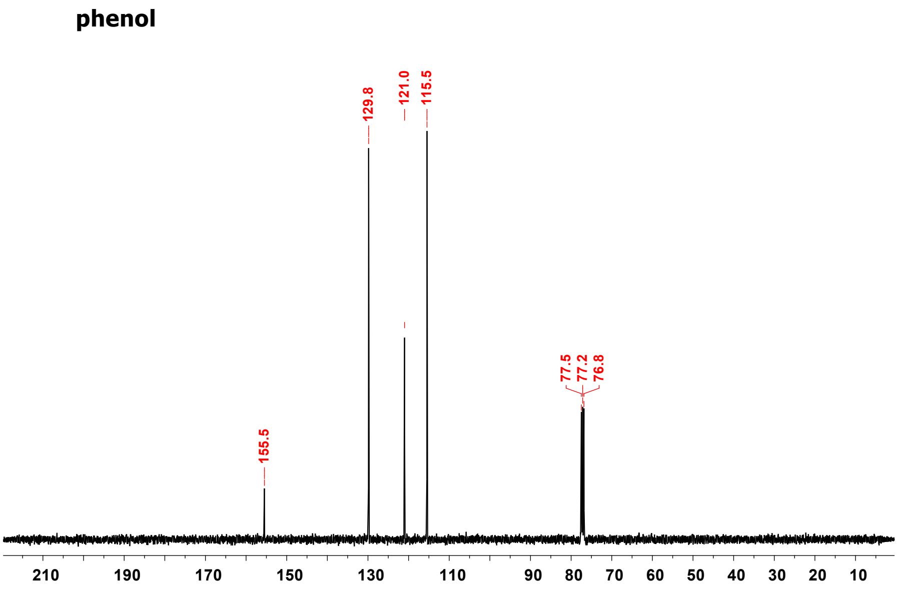

Figure S9: Phenol (2a). Isolated by column chromatography on silica gel using a mixture of ethyl acetate and pentane (1:9) as mobile phase to afford red solid in 86 % yield (8.1 mg). ^{1}H-NMR (400 MHz, $CDCl_3$): δ= 7.23 (t, *J*= 8 Hz, 2H), 6.93 (t, *J*= 7.4 Hz, 1H), 6.83 (d, *J*= 8 Hz, 2H). ^{13}C{^{1}H} -NMR (101 MHz, $CDCl_3$): δ= 155.5, 129.8, 121, 115.5.[1]

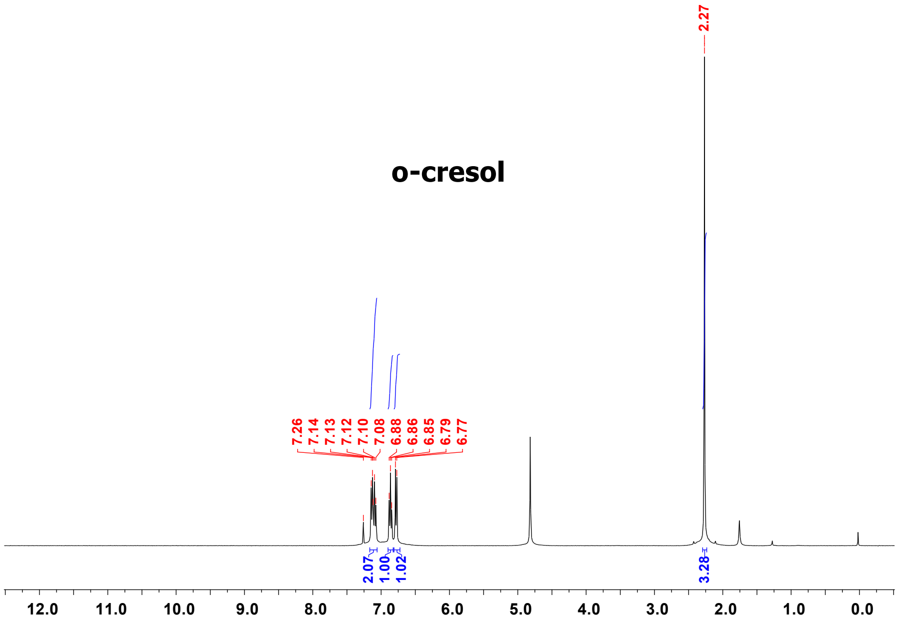

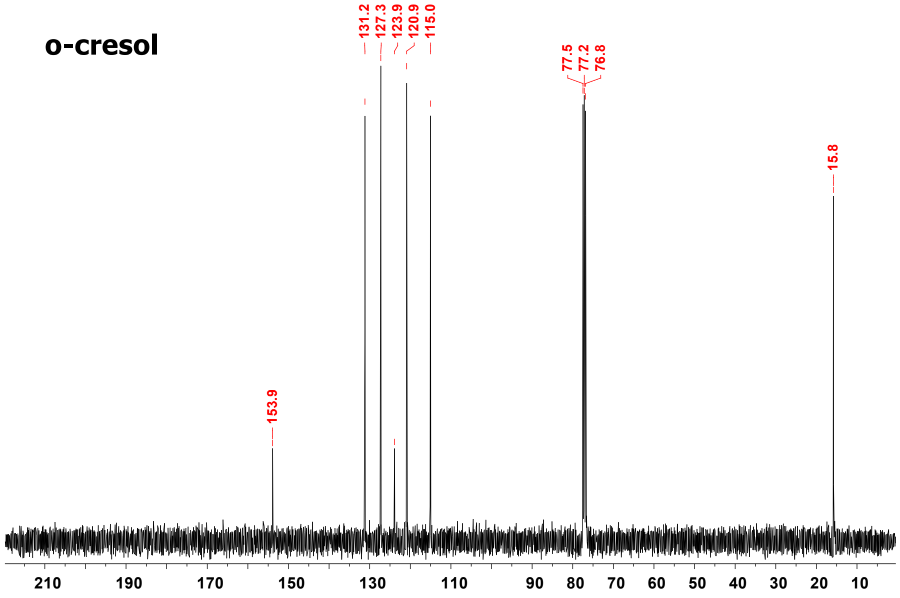

Figure S10: *o*-cresol (2b). Isolated by column chromatography on silica gel using a mixture of ethyl acetate and pentane (1:9) as mobile phase to afford colorless solid in a 90 % yield (9.7 mg). ^{1}H-NMR (400 MHz, $CDCl_3$): δ= 7.14 (d, J = 8 Hz, 1H), 7.10 (t, J = 7.8 Hz, 1H), 6.86 (t, J = 7.4 Hz, 1H), 6.78 (d, J = 8 Hz, 1H), 2.27 (s, 3H). ^{13}C{^{1}H}-NMR (101 MHz, $CDCl_3$): δ= 153.9, 131.2, 127.3, 123.9, 120.9, 115.0, 15.8.[2]

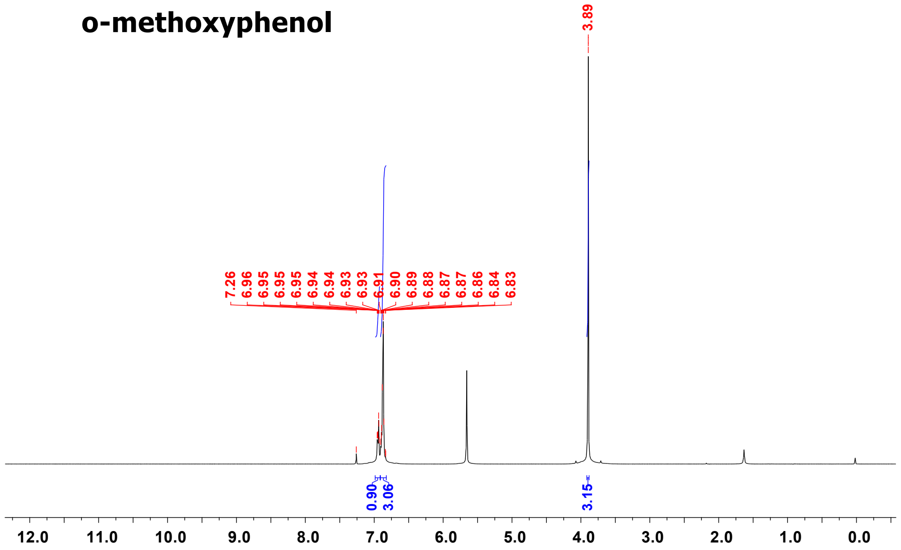

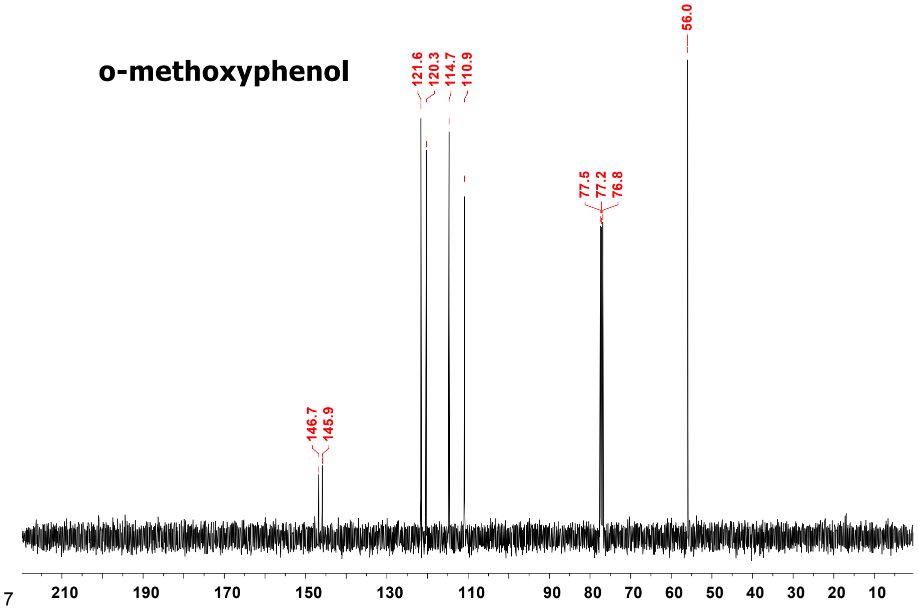

Figure S11: *o*-methoxyphenol (2c). quantified by ^{1}H-NMR and compared with an authentic sample. ^{1}H-NMR (400 MHz, $CDCl_3$): δ= 6.96–6.93 (m, 1H), 6.91–6.86 (m, 3H), 3.89 (s, 3H). ^{13}C{^{1}H}-NMR (101 MHz, $CDCl_3$): δ= 146.7, 145.9, 121.6, 120.3, 114.7, 110.9, 56.[3]

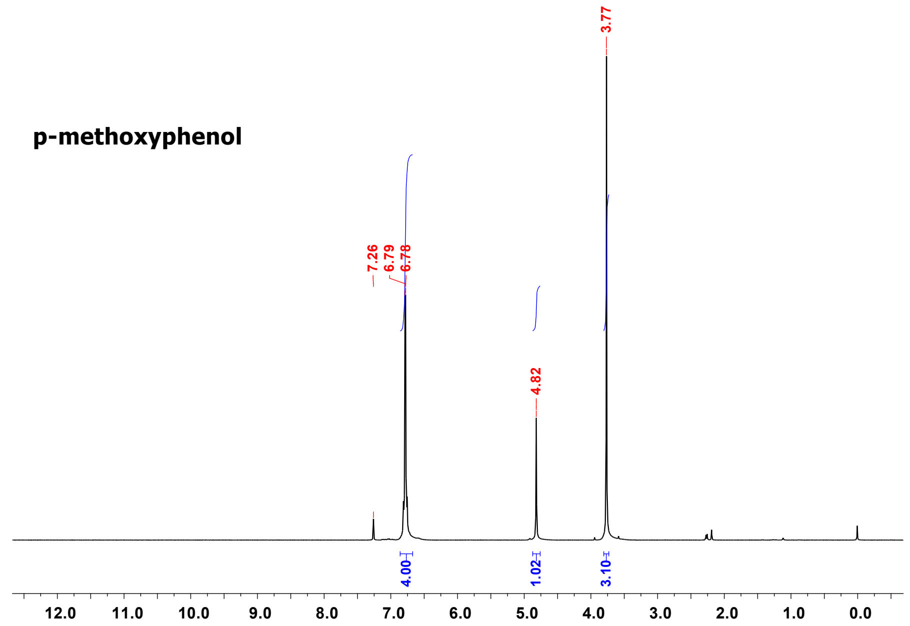

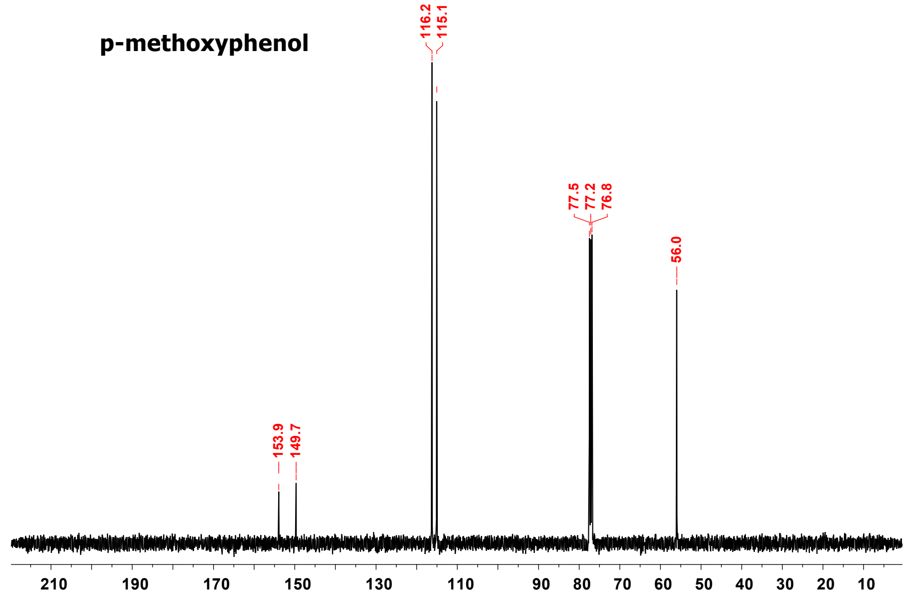

Figure S12: *p*-methoxyphenol (2d). Isolated by column chromatography on silica gel using a mixture of ethyl acetate and pentane (1:9) as mobile phase to afford white solid in a 75 % yield (9.3 mg).^{1}H-NMR (400 MHz, $CDCl_3$): δ= 6.79–6.78 (m, 4H), 4.82 (s, 1H), 3.77 (s, 3H). ^{13}C{^{1}H}-NMR (101 MHz, $CDCl_3$): δ 153.9, 149.7, 116.2, 115.1, 56.[1]

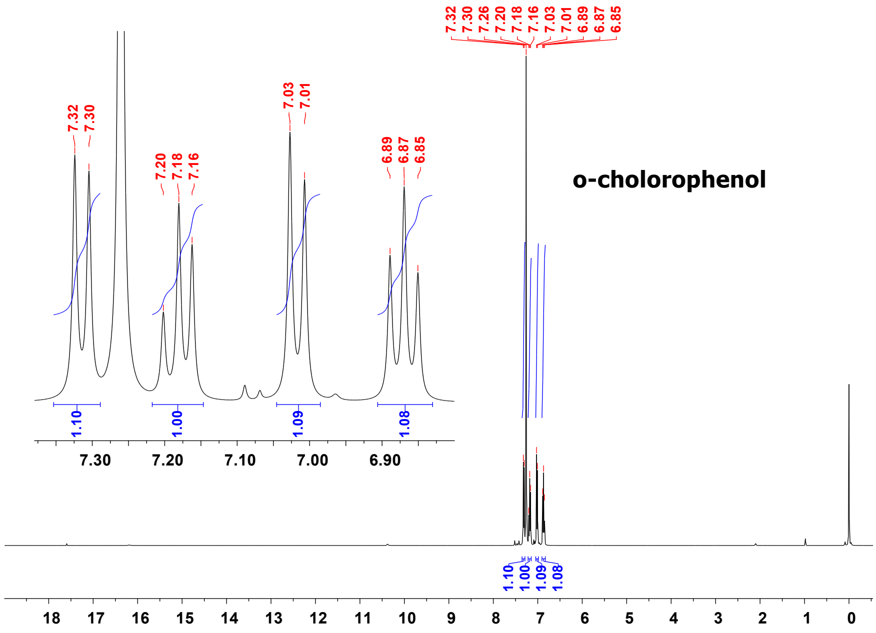

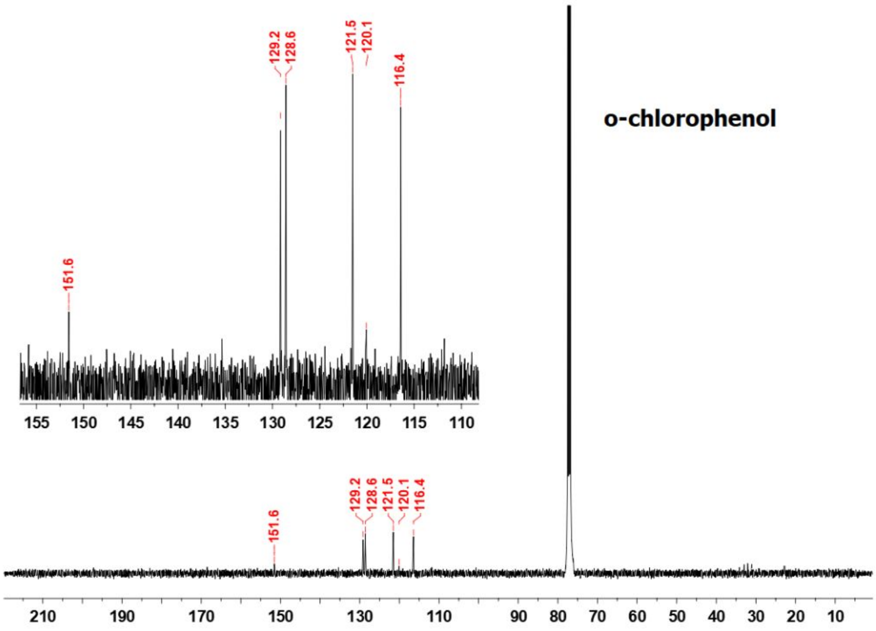

Figure S13: *o*-chlorophenol (2e). Isolated by column chromatography on silica gel using a mixture of ethyl acetate and pentane (1:9) as mobile phase to afford colorless oil in a 90 % yield (11.5 mg). ^{1}H-NMR (400 MHz, $CDCl_3$): δ= 7.31 (d, *J*= 7.8 Hz, 1H), 7.18 (t, J=7.5 Hz, 1H), 7.02 (d, *J*= 8 Hz, 1H), 6.87 (t, J = 7.8 Hz, 1H). ^{13}C{^{1}H}-NMR (101 MHz, $CDCl_3$): δ= 151.6, 129.2, 128.6, 121.5, 120.1, 116.4.[2]

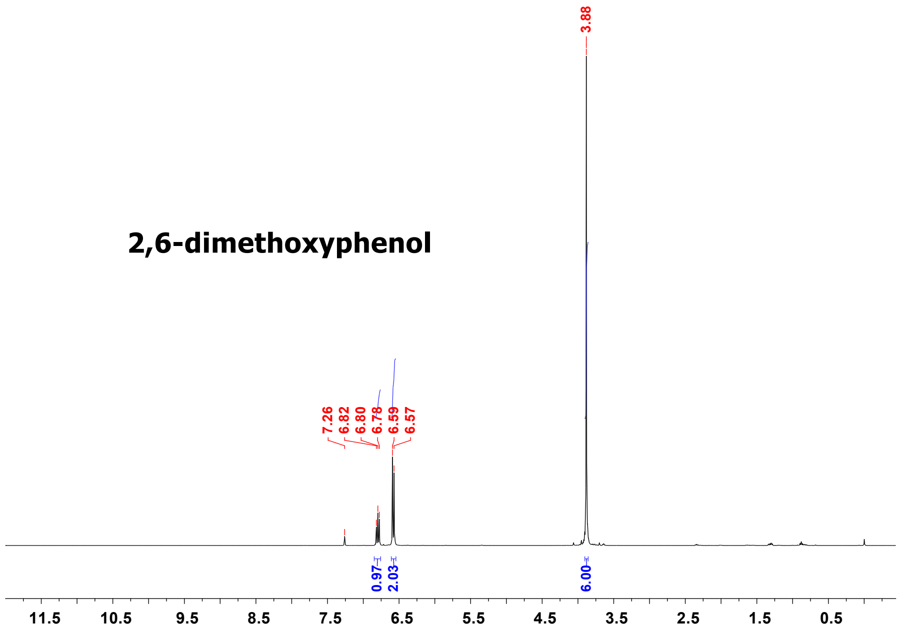

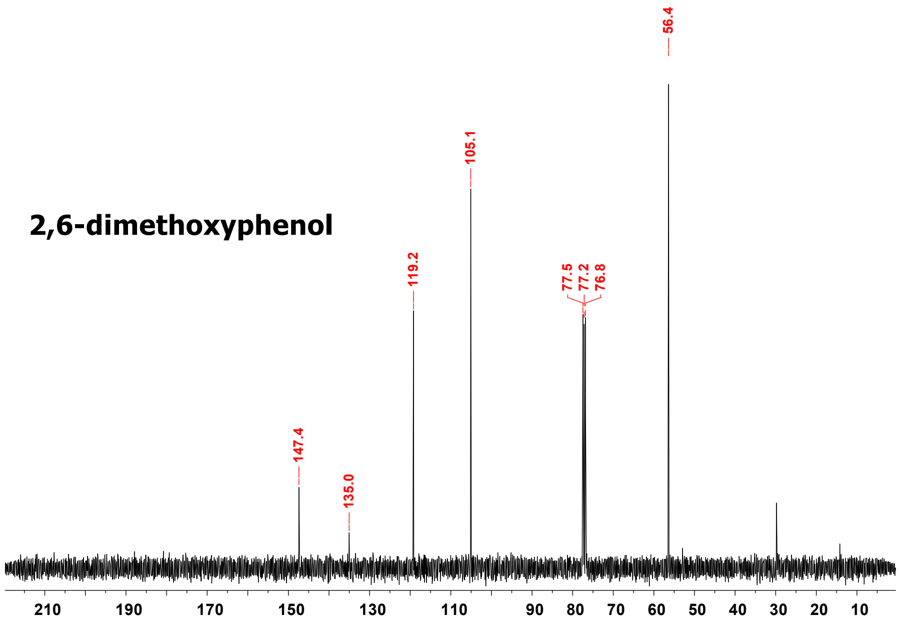

Figure S14: 2,6-dimethoxyphenol (2f). quantified by ^{1}H-NMR and compared with an authentic sample. ^{1}H-NMR (400 MHz, $CDCl_3$): δ= 6.80 (t, *J*= 8.4 Hz, 1H), 6.58 (d, *J*= 8.5 Hz, 2H), 3.88 (s, 6H). ^{13}C{^{1}H}-NMR (101 MHz, $CDCl_3$): δ= 147.4, 135.0, 119.2, 105.1, 56.4.[1]

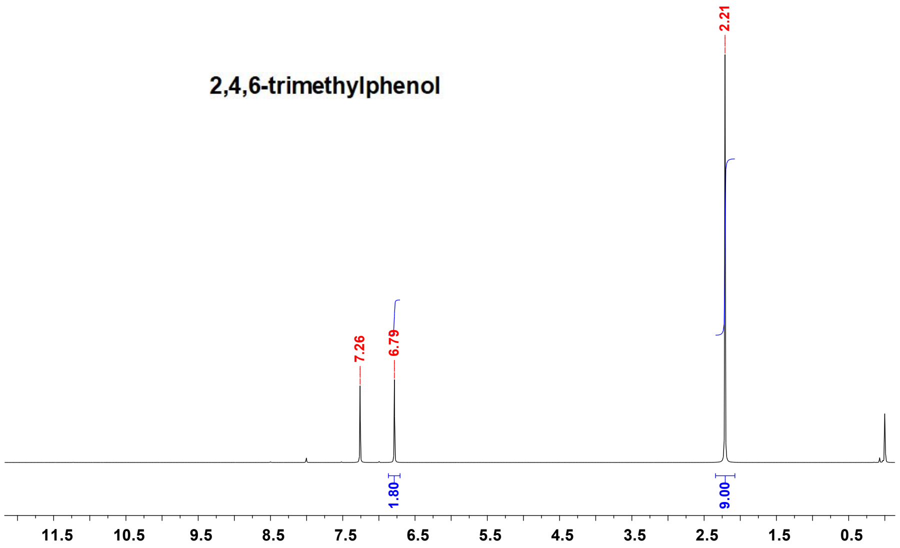

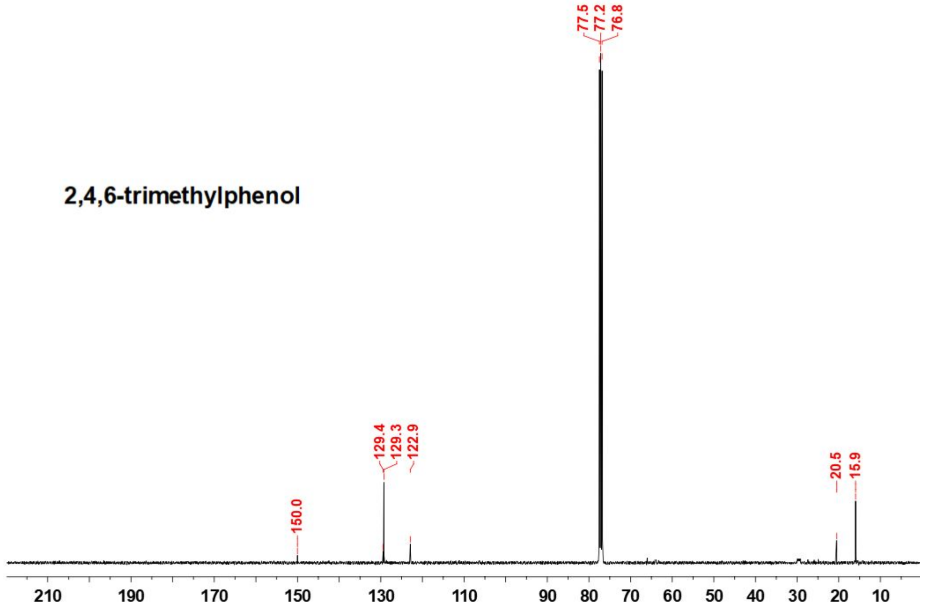

Figure S15: 2,4,6-trimethylphenol (2g). quantified by ^{1}H-NMR and compared with an authentic sample. ^{1}H-NMR (400 MHz, $CDCl_3$): δ= 6.79 (s, 2H), 2.21 (s, 9H). ^{13}C{^{1}H}-NMR (101 MHz, $CDCl_3$): δ= 150.0, 129.4, 129.3, 122.9, 20.5, 15.9.[3]

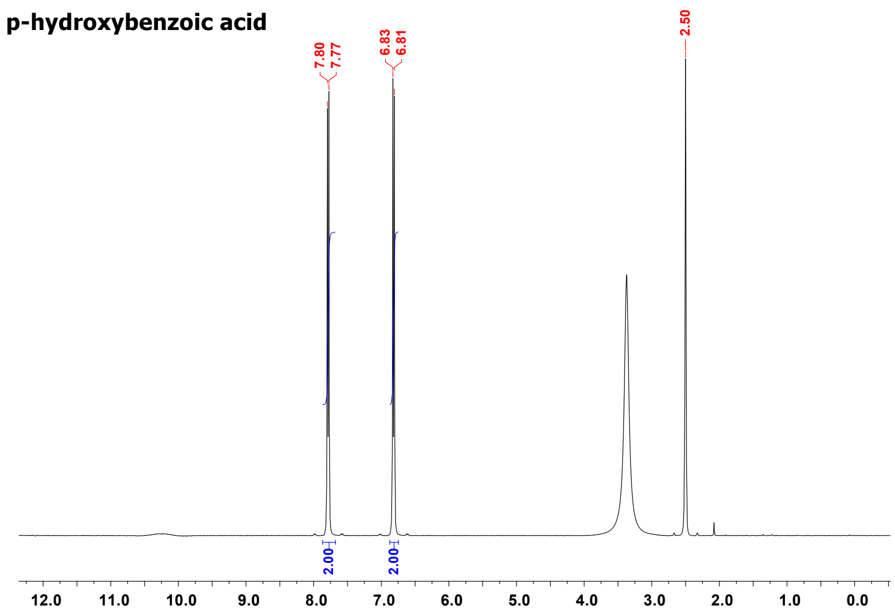

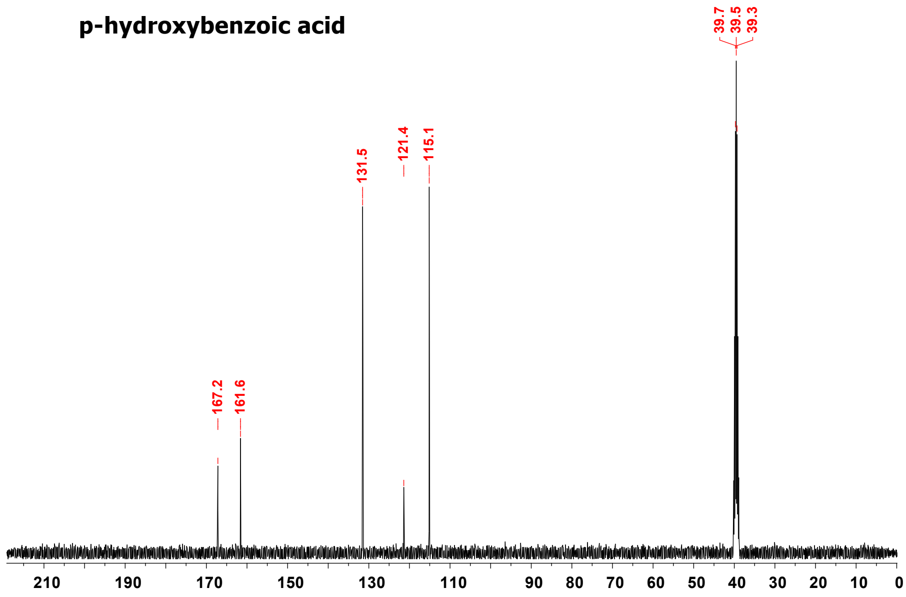

Figure S16: ***p*-hydroxybenzoic acid (2j)**. Isolated by column chromatography on silica gel using a mixture of ethyl acetate and pentane (1:1) as mobile phase to afford white solid in 85 % yield (11.7 mg).^{1}H-NMR (400 MHz, DMSO-d_6): δ= 7.78 (d, J = 8.6 Hz, 2H), 6.82 (d, J = 8.6 Hz, 2H). ^{13}C{^{1}H}-NMR (100 MHz, DMSO-d_6): δ= 167.2, 161.6, 131.5, 121.4, 115.1.[3]

Synthesis of phenylboronic acid MIDA ester (6-methyl-2-phenyl-1,3,6,2-dioxazaborocane-4,8-dione)

A reaction flask equipped with a magnetic stirring bar was charged with a mixture of 1.0 mmol phenylboronic acid (0.122 g), MIDA (3 mmol, 0.442 g) and molecular sieves (0.36 g) was dissolved in DMF (10 mL). The reaction mixture was heated at 120 °C under inert atmosphere and monitored by TLC. When the formation of MIDA boronate from phenylboronic acid was completed, the reaction mixture was cooled to room temperature and concentrated. The crude mixture was re-dissolved in acetone and filtered. The resulting MIDA boronate is soluble in acetone, whereas MIDA is not. The filtrate was purified by flash column chromatography on silica gel using acetone as eluent to afford a white solid. Then, the solid obtained was re-dissolved in a minimum of acetone to which Et_2O was slowly added to promote crystallization. MIDA boronate was collected by filtration as a white solid in a 90% reaction yield (0.210 g). The spectroscopic data are in good agreement with those reported in the literature.[4, 5]

Synthesis of Phenylboronic acid neopentylglycol ester (5,5-dimethyl-2-phenyl-1,3,2-dioxaborinane)

A reaction flask equipped with a magnetic stirring bar was charged with a 1.0 mmol of phenylboronic acid (0.122 g), neopentylglycol (2,2-Dimethyl-1,3-propanediol, 1.0 mmol, 0.104 g) and Et_2O (10 mL). The reaction mixture was stirred at room temperature and monitored by TLC. After complete consumption of phenylboronic acid, the solvent was removed under reduced pressure. The residue was purified by column chromatography on silica gel using a mixture of ethyl acetate and pentane (1:9) to afford compound **1l** as a white solid with a reaction yield >95 % (0.190 g). The spectroscopic data are in good agreement with those reported in the literature.[6]

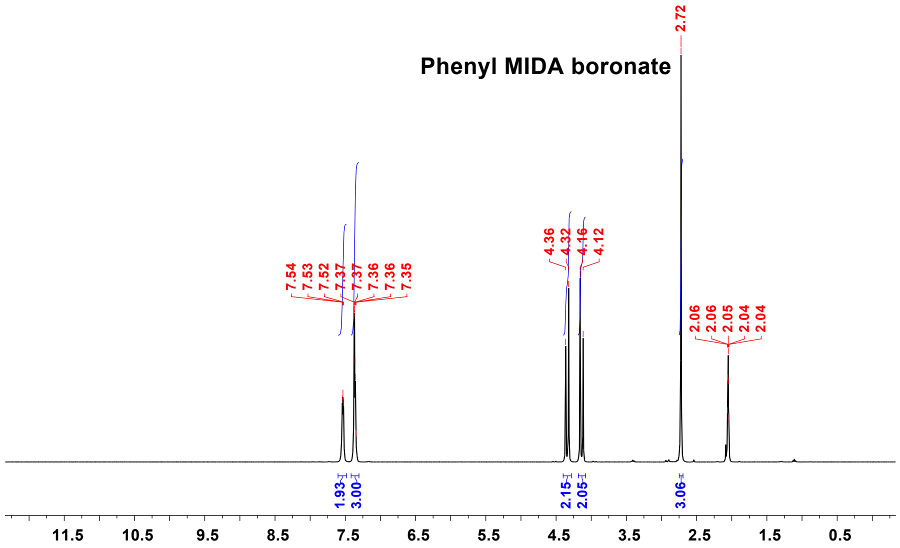

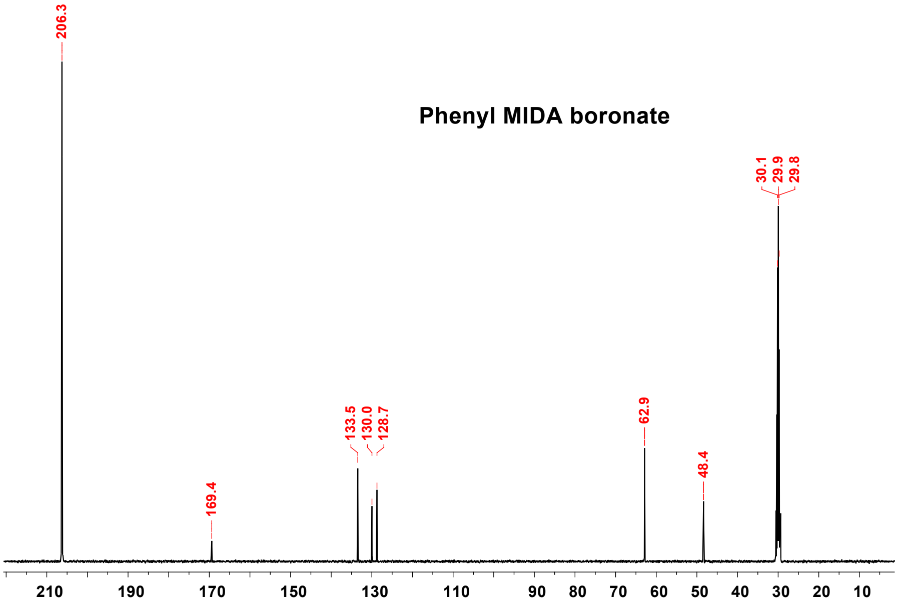

Figure S17: 6-methyl-2-phenyl-1,3,6,2-dioxozaborocano-4,8-dione (1k). ^{1}H-NMR (400 MHz, $(CD_3)_2CO$): δ= 7.52-7.54 (m, 2H), 7.35-7.37 (m, 3H), 4.34 (d, *J*= 17 Hz, 2H), 4.14 (d, *J*= 17 Hz, 2H), 2.72 (s, 3H). ^{13}C{^{1}H}-NMR (101 MHz, $(CD_3)_2CO$): δ= 169.4, 133.5, 130.0, 128.7, 62.9, 48.4.[4, 5]

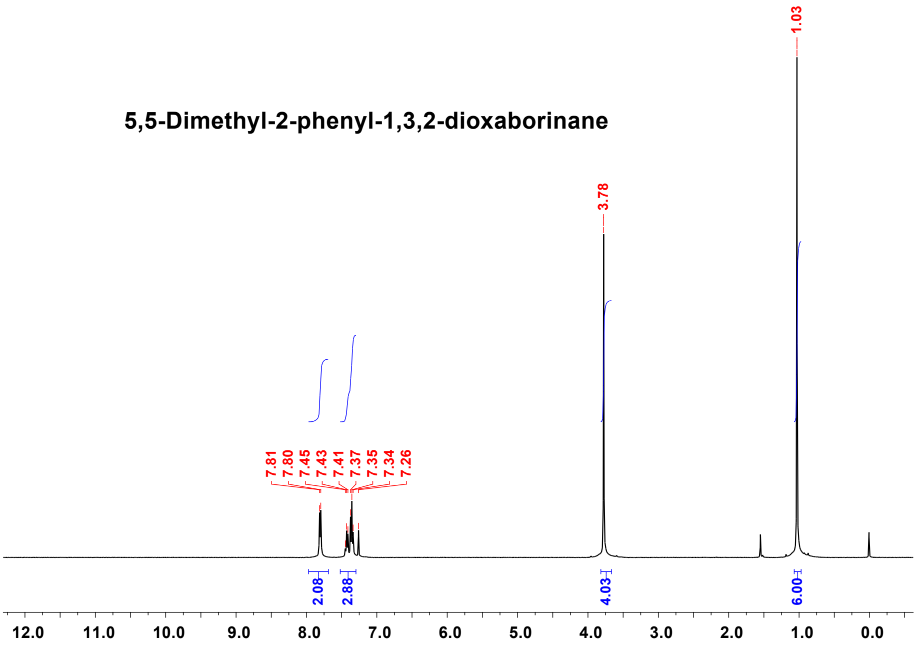

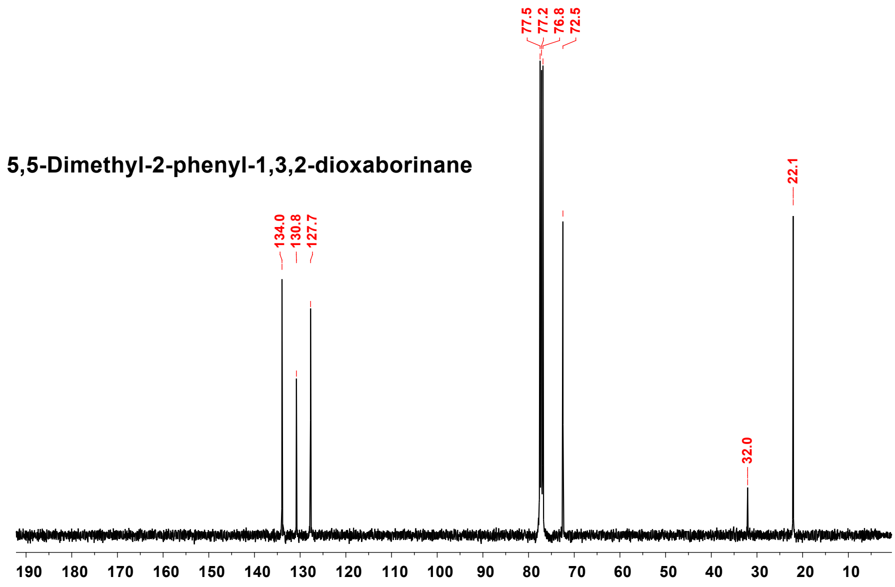

Figure S18: 5,5-dimethyl-2-phenyl-1,3,2-dioxoborinane (1l). ^{1}H-NMR (400 MHz, $CDCl_3$): δ= 7.80 (d, *J*=7 Hz, 2H), 7.43 (t, *J*= 7.2 Hz, 1H), 7.35 (t, J = 7 Hz, 2H), 3.78 (s, 4H), 1.03 (s, 6H). $^{13}C\{^{1}H\}$-NMR (101 MHz, $CDCl_3$): δ= 134.0, 130.8, 127.7, 72.5, 32.0, 22.1.[6]

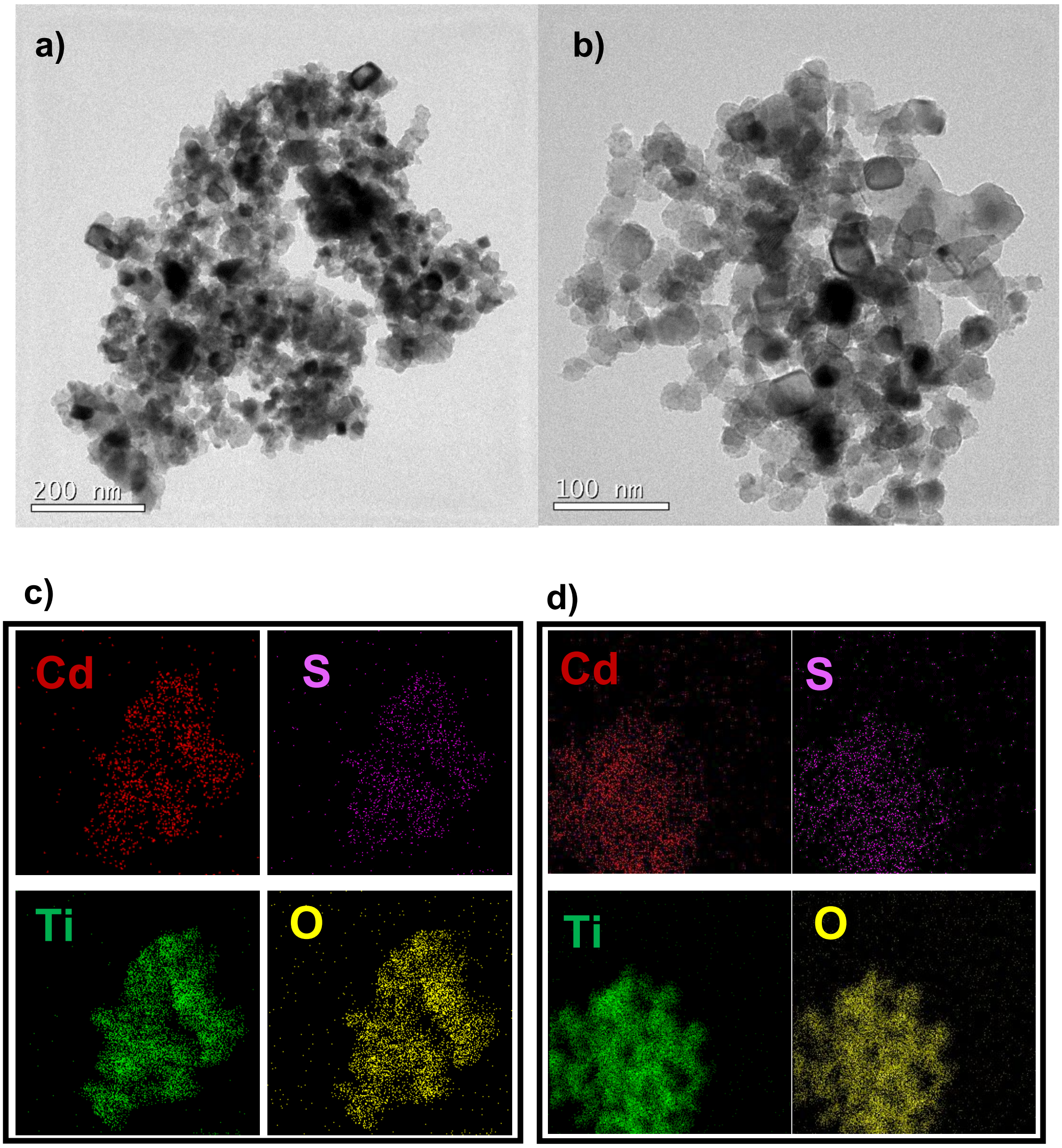

Figure S19: HRTEM images of the CdS@MPA-TiO2 hybrid a) before and after b) 5 photocatalytic cycles for the oxidative hydroxylation of 1a (scale bar = 200 and 100 nm, respectively). Energy dispersive X-ray spectroscopy (EDS) mapping analysis of the CdS@MPA-TiO2 hybrid c) before and d) after 5 photocatalytic cycles for the oxidative hydroxylation of 1a. Elements detected and homogeneously distributed in the material: Cd (red), S (pink), Ti (green) and O (yellow).

References:

1. Zhu, C.; Wang, R.; Falck, J. R., Mild and Rapid Hydroxylation of Aryl/Heteroaryl Boronic Acids and Boronate Esters with N-Oxides. *Organic Letters* **2012,** *14* (13), 3494-3497.
2. Xie, H.-Y.; Han, L.-S.; Huang, S.; Lei, X.; Cheng, Y.; Zhao, W.; Sun, H.; Wen, X.; Xu, Q.-L., N-Substituted 3(10H)-Acridones as Visible-Light, Water-Soluble Photocatalysts: Aerobic Oxidative Hydroxylation of Arylboronic Acids. *The Journal of Organic Chemistry* **2017,** *82* (10), 5236-5241.
3. Jiang, M.; Yang, H.-J.; Li, Y.; Jia, Z.-Y.; Fu, H., Metal-free synthesis of substituted phenols from arylboronic acids in water at room temperature. *Chinese Chemical Letters* **2014,** *25* (5), 715-719.
4. Close, A. J.; Kemmitt, P.; Emmerson, M. K.; Spencer, J., Microwave-mediated synthesis of N-methyliminodiacetic acid (MIDA) boronates. *Tetrahedron* **2014,** *70* (47), 9125-9131.
5. Baldwin, A. F.; North, R.; Eisenbeis, S., Trace Level Quantification of Derivatized Boronic Acids by LC/MS/MS. *Organic Process Research & Development* **2019,** *23* (1), 88-92.
6. Hu, J.; Zhao, Y.; Liu, J.; Zhang, Y.; Shi, Z., Nickel-Catalyzed Decarbonylative Borylation of Amides: Evidence for Acyl C−N Bond Activation. *Angew. Chem. Int. Ed.* **2016,** *55* (30), 8718-8722.
